# Genetic Variation Controlling Wrinkled Seed Phenotypes in *Pisum*: How Lucky Was Mendel?

**DOI:** 10.3390/ijms18061205

**Published:** 2017-06-06

**Authors:** Tracey Rayner, Carol Moreau, Mike Ambrose, Peter G. Isaac, Noel Ellis, Claire Domoney

**Affiliations:** 1 John Innes Centre, Norwich Research Park, Norwich NR4 7UH, UK; tracey.rayner@jic.ac.uk (T.R.); carol.moreau@jic.ac.uk (C.M.); mike.ambrose@jic.ac.uk (M.A.); 2 IDna Genetics Ltd, Centrum, Norwich Research Park, Norwich NR4 7UG, UK; peter.isaac@idnagenetics.com; 3 Department of Biology Sciences, University of Auckland, Auckland 1142, New Zealand; thnoelellis@gmail.com; 4 Department of Crop Physiology, International Centre for Agricultural Research in the Dry Areas (ICARDA), Rabat 10106, Morocco

**Keywords:** genetic markers, myoinositol, pea germplasm, *r* and *rb* mutations, seed phenotype, seed coat (testa) metabolites, wrinkled seeds

## Abstract

One of the traits studied by Mendel in pea (*Pisum sativum* L.) was the wrinkled-seeded phenotype, and the molecular basis for a mutation underlying this phenotype was discovered in the 1990s. Although the starch-branching enzyme gene mutation identified at the genetic locus *r* is most likely to be that in seeds available to Mendel in the mid-1800s, it has remained an open question as to whether or not additional natural mutations in this gene exist within *Pisum* germplasm collections. Here, we explore this question and show that all but two wrinkled-seeded variants in one such collection correspond to either the mutant allele described previously for the *r* locus or a mutation at a second genetic locus, *rb*, affecting the gene encoding the large subunit of Adenosine diphosphoglucose (ADP-glucose) pyrophosphorylase; the molecular basis for the *rb* mutation is described here. The genetic basis for the phenotype of one (JI 2110) of the two lines which are neither *r* nor *rb* has been studied in crosses with a round-seeded variant (JI 281); for which extensive genetic marker data were expected. In marked contrast to the trait studied by Mendel and the *rb* phenotype; the data suggest that the wrinkled-seeded phenotype in JI 2110 is maternally determined, controlled by two genetic loci, and the extent to which it is manifested is very sensitive to the environment. Metabolite analysis of the cotyledons of JI 2110 revealed a profile for sucrose and sucrose-derived compounds that was more similar to that of wild-type round-seeded, than that of wrinkled-seeded *r*, pea lines. However, the metabolite profile of the seed coat (testa) of JI 2110 was distinct from that of other round-seeded genotypes tested which, together with analysis of recombinant inbred progeny lines, suggests an explanation for the seed phenotype.

## 1. Introduction

Pulse and other legume crops play an important role in agriculture as a sustainable source of protein, starch, and other nutrients. When grown in crop rotations, or as intercrops, they reduce the fertiliser demand because of their nitrogen-fixing properties and concomitant increase in soil organic matter, maintaining soil fertility. Seed quality traits are extremely important in determining the market value of pulses and therefore return to growers, which in turn influence crop choices in rotations (where often the second crop advantage is not properly accounted). In attempting to maximise the incorporation of legume crops into rotations, an understanding of the genetic basis for important end-use quality traits is crucial to the development of new varieties that meet market requirements and provide incentives to growers. One such trait of importance to the vegetable industry is sweetness, linked in many crops including *Pisum sativum* L. (pea) to genetic perturbations in starch biosynthesis [1,2,3].

Mutations which lead to higher concentrations of sugars in pea seeds include the naturally occurring mutation leading to the wrinkled-seeded phenotype studied by Mendel [4]. The molecular genetic basis for this mutation (*r*) is now known to be a consequence of an insertion in the *SbeI* gene, affecting the carboxy-terminal region of the starch-branching enzyme, which is responsible for the synthesis of amylopectin in developing pea seeds [1]. This defect leads to direct and pleiotropic effects in seeds, including higher concentrations of sucrose and sucrose-derived metabolites, a starch that is amylose-rich, higher seed protein concentrations with a reduced content of the major storage protein, legumin, lower moisture content, and reduced seed longevity [5,6,7]. Nonetheless, the mutation has been widely adopted by the vegetable industry and is the basis for most commercial vining cultivars.

There is now a renewed interest in the nature of such perturbations to plant starch biosynthesis and the types of starch that result. While pulses and other legume crops are generally accepted to be an invaluable part of a healthy balanced diet, they have also been implicated in the prevention of chronic-degenerative diseases such as certain types of cancer, heart disease, and diabetes. For the prevention of Type II diabetes, starch that is more resistant to digestion offers benefits to human health, linked to positive effects on short-chain fatty acid and pancreatic signalling and blood glucose [8,9]. High amylose starch is particularly desirable in conferring these benefits [9].

Mutations affecting additional enzymes of the starch biosynthetic pathway can also lead to seed wrinkling phenotypes, but the starch-branching enzyme (*sbeI*) gene mutation identified at the genetic locus *r* is most likely to be that in the variant lines of pea available to Mendel in the mid-19th century [1]. Another wrinkled-seed mutation, at the *rb* locus, affects the gene encoding the large subunit of ADP-glucose pyrophosphorylase [10], but this mutation has only become available in Europe since the 1930s [11,12]. It has remained an open question, however, as to whether or not additional natural mutations in these and other starch metabolism genes exist within *Pisum* germplasm collections. One such collection of over 3000 lines has been shown to be comprised of a number of related sub-populations, based on analysis of kinship and shared markers [13]. The collection has been shown to contain a rare mutant allele of *A*, controlling anthocyanin pigmentation of flowers, in addition to the mutant allele responsible for most white-flowered variants and likely to have been the allele Mendel studied [4,14]. High-throughput screening of the collection has identified one individual mutant line (a *Pisum elatius* accession) which lacks seed trypsin-chymotrypsin inhibitors, also demonstrating the value of the resource [15]. Here we utilise the germplasm collection and screening methods to investigate whether or not accessions with wrinkled-seeded phenotypes can be explained by mutations identified previously. We provide new data and insights related to *r* and *rb* mutations and describe novel variation in seed phenotype, which appears not to be simply inherited and is maternally controlled.

## 2. Results

### 2.1. Germplasm Screen for sbeI Variants

The germplasm screen was based on 2792 *Pisum* accessions, representing the broad genetic diversity available across the JIC *Pisum* accessions (Section 4.1; File S1). The genetic screening of DNA from this collection has been described previously [13,14,15] and has demonstrated the utility of the resource in identifying rare alleles of known genes [14,15].

**Figure 1:**
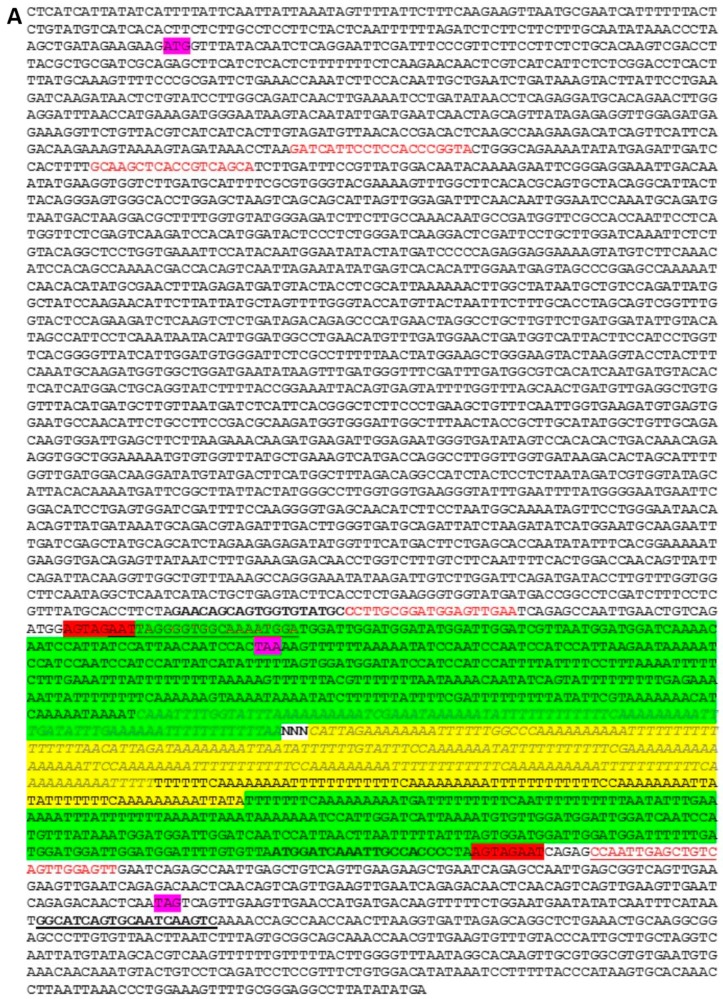

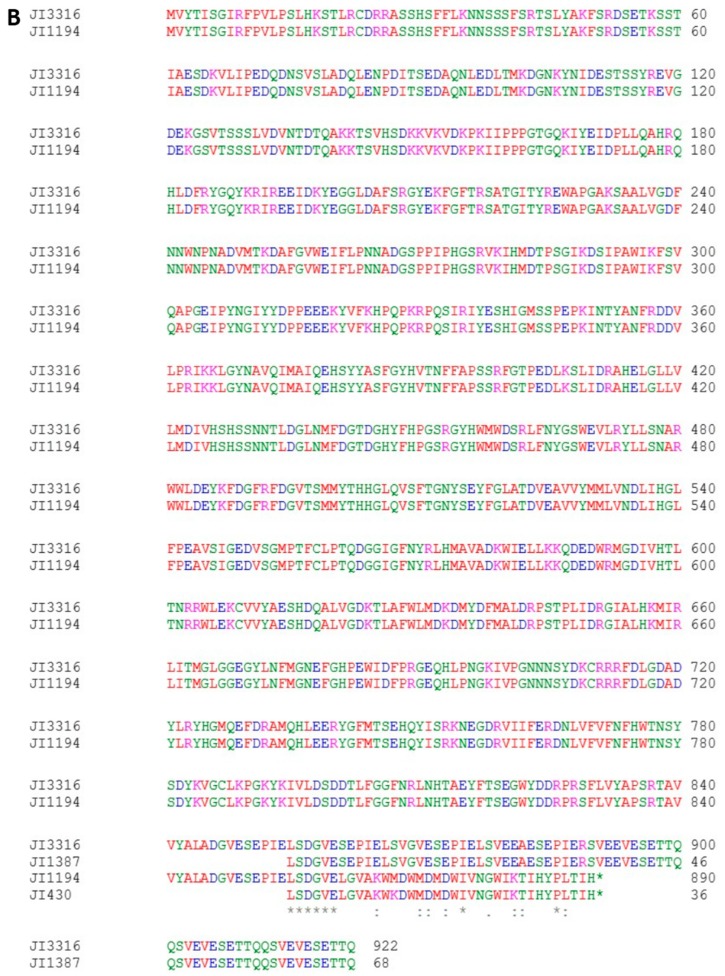
(**A**) Manual assembly of contigs obtained by sequencing cDNA corresponding to *sbeI* in the wrinkled-seeded pea accession, JI 1194, allows the insertion sequence to be partly determined. The colour codes for the highlighted regions are given in the key. For designing primers specific to the insertion mutation in *r* mutants, the sequence highlighted in green was used. The positions of primer sequences are indicated in red/bold font, with reverse primer sequences underlined. The positions of primers used to provide a facile diagnostic test for *RR* vs. *rr* genotypes are shown in bold font. Diagnostic primers used in the high-throughput genetic screen of germplasm to distinguish *RR* from *rr* genotypes are shown in red font; KEY: GTCAGATG = wild-type sequence (round-seeded lines); AGTAGAAT = 8bp flanking insertion; GTAAAATA = *r* insertion sequence confirmed; AAAAATTC = good quality *r* insertion sequence, unconfirmed; ATTAGATAATTAGATA = poor quality *r* insertion sequence, unconfirmed; CAAAAATACAAAAATA = poor quality *r* insertion sequence unconfirmed; ---- = earlier and later sequence not forming a contig; ATG = initiator methionine; TAG/TAA = stop codon in wild type, deduced in mutant; Primer sequences are indicated in red/bold font, with reverse primer sequences underlined; (**B**) The SBEI/sbeI protein sequences deduced for a *sbe1* mutant variant (JI 1194) in comparison with a wild type (JI 3316). The protein predicted for the mutant diverges from that of the wild type towards the carboxy-terminal region. The variation was validated by partial sequences determined for a second mutant line, JI 430, in comparison with a further wild type line, JI 1387. Amino acids are colour-coded: negatively charged, blue; positively charged, magenta; C, G, H, N, Q, S, T, Y, green; all other amino acids, red. Identities and similarities are indicated for the divergent mutant and wild-type carboxy-terminal sequences (*, identical; :, very similar; ., similar, amino acids).

A naturally occurring mutation in the pea *sbeI* gene was described previously as a consequence of an insertion event within the 3′ region of the coding sequence, disrupting the carboxy-terminal region of the encoded protein [1]. The insertion is flanked by a repeat sequence and has the hallmark of an *Ac/Ds*-like transposable element. No other naturally occurring *sbeI* mutation has been described for pea, and the insertion mutation is believed to be the basis for the wrinkled-seeded phenotype studied by Mendel [4] and at the *r* locus. The appearance of starch grains is also affected by the mutation, and compound starch grains are a feature of the natural and induced mutations at the *r* locus [2,16]. Here, we assay 2792 lines from the JIC *Pisum* germplasm collection for *sbeI* and wrinkled-seeded variants. Five pairs of primers were designed to identify *sbeI* variants, three pairs of which were deployed for the germplasm multiplex screen, one pair (Ps-SBEI-F4/Ps-SBEI-R4) for simplex PCR assays and a further primer to act in combination with Ps-SBEI-F4/Ps-SBEI-R4 as a triplex PCR (Section 4.1). Multiplex PCR assays were designed to yield amplicons which could be identified both by fluorescent label (either Fam, blue, or Vic, green) and size (within the range of 50–300 bp; see image in File S1). In the multiplex assays, Sbe1 F4F/R4e (see Section 4.1) amplified a fragment upstream of the *r* insertion site, which served as a control amplicon but would also identify insertion/deletion variants within this region had they existed. Two other primer sets were designed to determine if the insertion mutation was present (Sbe1 rcFV/rIR) or absent (Sbe1rcFV/rDR). The positions of the primers in relation to the *sbeI* mutation are shown in Figure 1A, which gives the sequence of the insertion that could be assigned with confidence; the protein predicted by the mutant gene (Figure 1B) is truncated at the carboxy-terminal relative to wild type, which shows a repetitive sequence. This, and the very repetitive nature of the insertion element, severely limited the extent to which primers could be designed in these regions, and also hindered amplification and determination of end-to-end sequence for the insertion element (Figure 1A).

**Figure 2:**
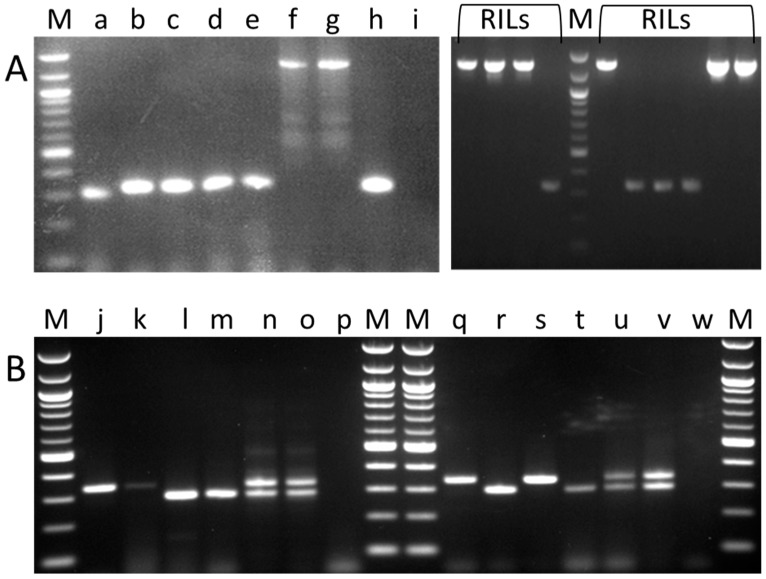
Diagnostic PCR tests for variant wild-type (*R*) and mutant *sbeI* (*r*) alleles. (**A**) Simplex PCR assays showing a 340 bp fragment, derived from the wild-type gene, and a ~1250 bp fragment, representing the mutant allele. DNA was assayed from (a) JI 2202, *RR*; (b) JI 2822, *RR*; (c) JI 15, *RR*; (d) JI 399, *RR*; (e) JI 281, *RR*; (f) JI 1194, *rr*; (g) JI 1201, *rr*; (h) cv. Princess, marrowfat; (i) control PCR (lacking DNA). The right hand panel shows assays of recombinant inbred lines (RILs) derived from a cross between JI 15 and JI 1194; (**B**) Triplex PCR assays showing the same wild-type amplicon as shown in (**A**), with a smaller (298 bp) amplicon derived from the mutant (insertion to 3′ non-coding) allele, enabling heterozygotes to be scored readily. DNA was assayed from (j,k) JI 1417; (l,m) JI 1194; (n) JI 1417 × JI 1194 F1; (o) JI 1194 × JI 1417 F1; (p) control PCR (lacking DNA); (q) JI 15; (r) JI 1194; (s) JI 15; (t) JI 1194; (u,v) JI 15 and JI 1194 DNA mix; (w) control PCR (lacking DNA). Tracks labelled M show DNA markers (100 bp ladder, New England BioLabs^®^, Ipswich, MA, USA).

For simplex PCR analysis of the *sbeI* insertion (*sbeI-ins*) mutation, an assay was designed that amplified a fragment of approximately 340 bp in wild-type (*SbeI-wt*) but ~1250 bp in mutant lines (Figure 2A). This assay allowed the validation or otherwise of the germplasm screen results and also facilitates the rapid diagnosis of the presence or absence of this mutation in germplasm stocks, for which phenotype data may be absent or misleading. A modification to this assay using three primers was also developed and, though not used routinely in this work, is presented here as an assay suitable for identifying heterozygous lines with greater confidence, due to the similar sizes of amplicons generated for mutant and wild-type alleles (Figure 2B).

In the germplasm screens, no size variants were apparent for the *SbeI* F4F/R4e amplicon. The multiplex assay identified three classes of allelic composition: (i) lines carrying only the *SbeI-wt* allele; (ii) lines carrying only the *sbeI-ins* allele; and (iii) lines for which both alleles were detected. The first two were interpreted as homozygous lines and the third class was assumed to correspond to heterozygotes as the DNA had been prepared from single individuals [13]. These results were checked against the phenotypic data that were available for the germplasm collection (round/wrinkled/marrowfat seed appearance, where marrowfat denotes a classification of large seeds with irregular shape; File S1; [13]). The lines where the phenotype was not as predicted by the allelic composition of *SbeI* were investigated further, initially by phenotyping available seed and then examination of progeny seeds, with simplex PCR analysis of *SbeI* (Ps-SBEI-F4/Ps-SBEI-R4 primers) and *AgpL* (Section 2.2) genes.

Table 1 presents a summary of the numbers of germplasm accessions in the different phenotype groupings, compared with their *SbeI* genetic scores, based on the multiplex screen. The results suggested that 12 lines appeared to be heterozygous (Table 1). Phenotypic analysis suggested that four were indeed heterozygotes, whereas the others were true breeding, yielding either round or wrinkled seeds (four lines each). Genotypic analysis confirmed the allelic status of the presumed heterozygotes and showed the four round-seeded lines to be *SbeI-wt* homozygotes. Of the four wrinkled-seeded lines, three were homozygous for the mutant (*sbeI-ins*) allele. The remaining line (JI 2012) carried a wild-type *SbeI* allele and was therefore investigated alongside other lines in this group as to the status of its *AgpL* (Section 2.2) gene.

Among the “Round/Dimpled” accessions (Table 1), 42 lines were scored as carrying the *sbeI-ins* allele in the multiplex screen. Following re-analysis of the 42 lines, it was clear that their seed phenotypes were ambiguous and they were reassigned as 28 round, 6 dimpled, and 8 wrinkled and consistent with the assay. Re-screening these lines for their *SbeI* allele showed that the 28 round-seeded lines were in fact homozygous for *SbeI-wt*. The eight wrinkled-seeded lines, and all dimpled-seeded lines bar one (tg_306), were scored as homozygous for *sbeI-ins*. The line tg_306 was genotyped as *SbeI-wt*, indicating that the “dimpled-seeded” phenotype category contains *SbeI-wt* as well as *sbeI-ins* genotypes; tg_306 was shown to be wild-type for *AgpL* (Section 2.2) with simple starch grains (see starch grain morphology below).

Analysis of the 28 marrowfat lines which scored as homozygous for the *sbeI-ins* allele (Table 1, one line of 29 did not germinate) revealed that 14 were in fact “marrowfat” lines and the remainder were misclassified: 11 lines were wrinkled, 2 dimpled, and 1 was round. For the majority of the marrowfat lines which scored as homozygous for *sbeI-ins* in the initial germplasm screen, the scores were validated on re-screening. The line re-classified as round was shown to be *SbeI-wt*, while the two with the dimpled classification were found to be *sbeI-ins*. The data overall are consistent with most “marrowfat” lines being wild type for *SbeI* with a smaller proportion of this group having the mutant *sbeI* allele, and so the “marrowfat“ market class may be genetically either round or wrinkled.

Among the “wrinkled-seeded” category (Table 1), 59 lines were scored as *SbeI-wt* and this group could in theory yield novel mutations at *r* or at additional genetic loci affecting seed phenotype. Re-phenotyping 58 of these lines (one did not germinate) showed that five were round-seeded and two dimpled-seeded (JI 1599 and JI 2362, see below). Simplex assays for *SbeI* genotype revealed that 37 of the wrinkled-seeded lines were in fact homozygous for *sbeI-ins* and confirmed that the round- and dimpled-seeded lines were *SbeI-wt* homozygotes. Therefore, 14 lines remained from this analysis as having wrinkled seeds but being *SbeI-wt*. Thus unravelling a combination of failures within the multiplex analysis, plus errors in available phenotype data, revealed that overall 15 (14, plus JI 2012 above) lines could be classified as wrinkled-seeded but lacking the *sbeI* insertion mutation. These 15 lines were analysed for mutations in other genes affecting the starch biosynthetic pathway.

The appearance of starch grains was used to add confidence to the elimination of lines as being novel *sbeI* variants. For example, the two dimpled-seeded lines (JI 1599 and JI 2362) which were classified as “wrinkled-seeded” but lacked the *sbeI* insertion were shown to have simple, rather than compound, starch grains (see later, Section 2.3). Compound starch grains are expected for *sbeI* mutations [2,16].

### 2.2. Screening for the AgpL Mutation

**Figure 3:**
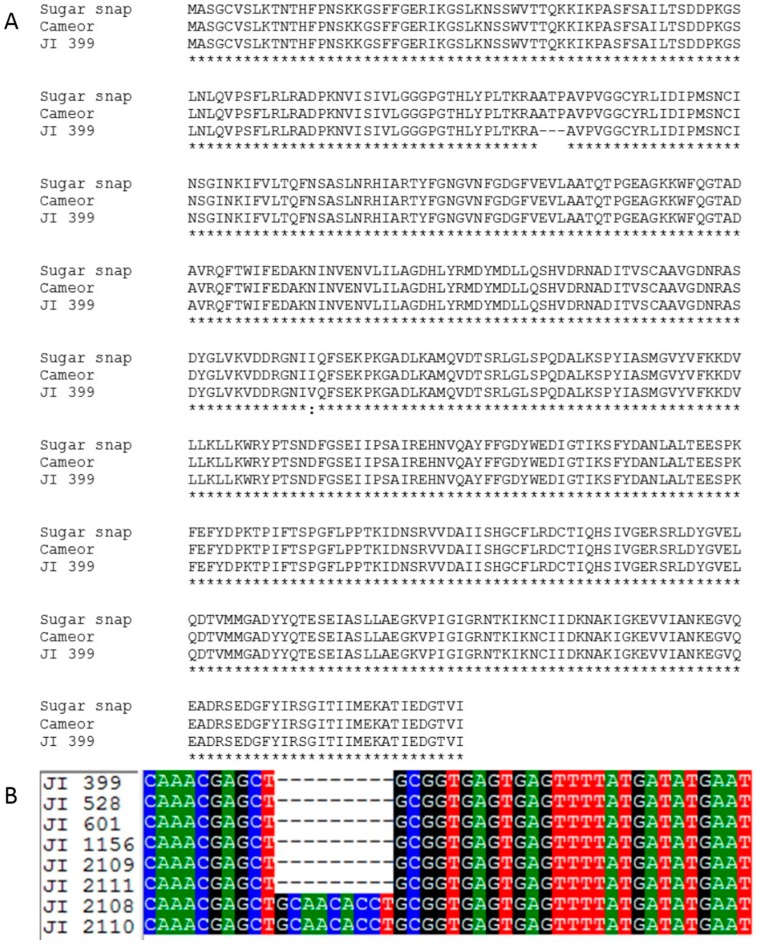
(**A**) Comparison of the mutant protein predicted by the *agpl* gene sequence in JI 399, a *rb* mutant lacking three amino acids (ATP) compared with the wild-type protein predicted for the cultivars Cameor and Sugar snap, the last being the source of the database NCBI submission X96766; asterisks indicate amino acid identity; (**B**) Deletion of the region in the *agpl* gene sequence of six germplasm accessions, all of which have a wrinkled-seeded phenotype, in comparison to the wild-type gene in two accessions. The JI accession numbers are indicated on the left of the corresponding sequences. Nucleotides are colour-coded: C, blue; A, green; G, black; T, red. (Note that intron 2 begins after the deletion, with the nucleotides GT).

A second naturally occurring mutation affecting seed shape in pea was described some decades ago and this locus became known as *rb* [2,5,11,17]. Biochemical studies of *rb* mutants indicated a lesion affecting the activity of the large subunit of the enzyme ADP-glucose pyrophosphorylase (AgpL) [10], but the molecular lesion underlying this mutation was not described. Analysis of the *agpl* gene from JI 399 (*rb*) revealed a nine base pair deletion in the coding region of the gene just prior to the donor splice site of intron 2 (Figure S1; GenBank accession number MF186819), leading to a loss of three amino acid residues (ATP) from the predicted protein (Figure 3A).

Primer pairs covering the *agpl* deletion were used to amplify and sequence the genic region from the 15 wrinkled-seeded lines which lacked the *sbeI* insertion mutation (Section 2.1). Of these, 13 lines showed the same nine bp deletion as shown for JI 399, identifying two lines, JI 1417 and JI 2110, as having a wrinkled-seeded phenotype but which lack either the *sbeI* insertion or the *agpl* deletion mutations (Table 2). The deletion of the ATP-encoding region in some *rb* lines, compared to JI 2110 and a *sbeI* mutant (JI 2108) is shown in Figure 3B. The combined germplasm analyses indicate that the wrinkled-seeded lines that are *r* mutants all carry the *sbeI-ins* mutant allele, all *rb* mutants carry the nine bp deletion allele of *agpl*, and two germplasm lines, JI 1417 and JI 2110, are novel naturally occurring genetic variants for seed shape.

### 2.3. Allelism Tests of JI 2110, A Novel Wrinkled-Seeded Mutant

Of the two wrinkled-seeded lines (JI 2110, JI 1417) which are neither *r* nor *rb*, JI 2110 was chosen for detailed genetic analysis. Allelism tests were carried out to discover whether the mutation in JI 2110 was at a locus known to influence starch metabolism, but distinct from *r* and *rb*. Three additional genetic loci have been described, based on the characterisation of induced mutations in pea which lead to a wrinkled-seeded phenotype [2,5]. The genes affected are phosphoglucomutase (*rug 3* locus) [18], sucrose synthase (*rug 4* locus) [19] and granule-bound starch synthase II (*rug 5* locus) [20]. Preliminary analysis of JI 1417 suggested that this line behaved in a similar way to JI 2110 with respect to maternal determinism of the testa trait (not shown). However, allelism tests between JI 1417 and JI 2110 were inconclusive; the analyses were confounded by JI 1417 having an extremely short habit (likely *na*, affecting the gibberellic acid pathway), few flowering nodes and square-shaped seeds. Therefore, the majority of the further genetic analyses were carried out on JI 2110.

**Figure 4:**
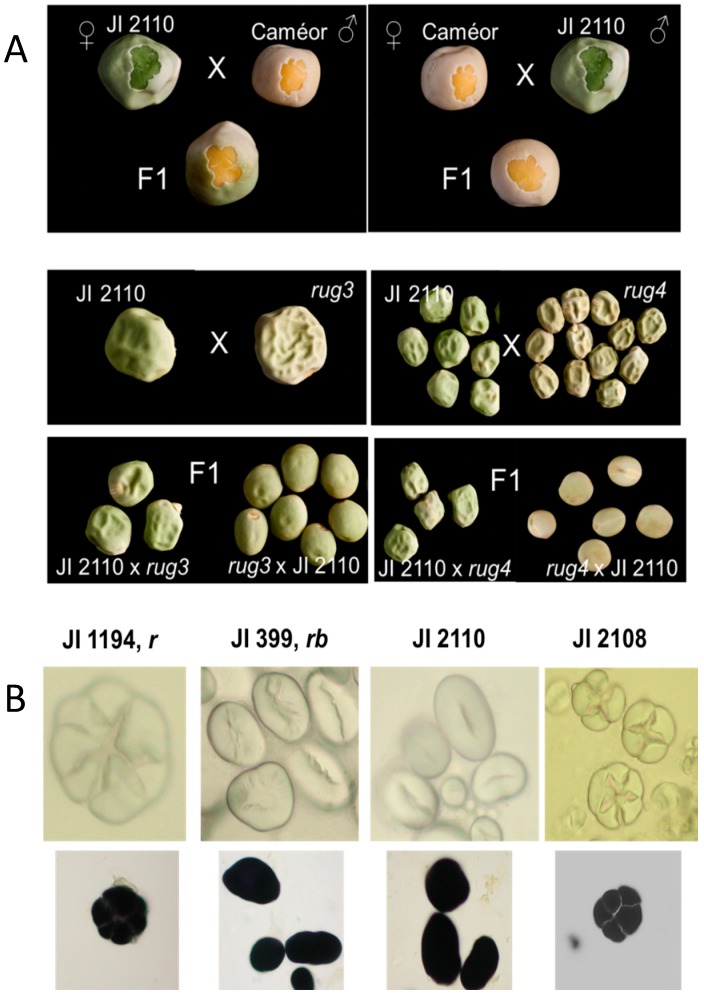
(**A**) Allelism tests of JI 2110 crossed to the wild-type round-seeded control line (cv. Cameor, top two panels) and to the wrinkled-seeded lines with mutations at *rug3*, *rug 4* (lower four panels). The female parent is listed first for every cross. Individual or groups of F1 seeds shown underneath the corresponding cross; (**B**) Images of starch grains from *r* (JI 1194, compound) and *rb* (JI 399, simple) control lines, JI 2110 (simple starch grains) and a further germplasm accession (*sbeI -ins*, *r*) with compound starch grains. The lower panel shows starch grains from the four lines stained with iodine. Various magnifications were used to emphasise the differences in shape of starch grains; bar scales not shown.

Reciprocal crosses were carried out between JI 2110 and lines carrying mutations at *r*, *rb*, *rug 3* and *rug 4*, in addition to the wild-type JI 3253 (cultivar, cv. Cameor). The status of the F1 seeds and plants generated was supplemented by sequence analysis of the gene affected by the test mutation and, where appropriate, by starch grain morphology and cotyledon colour (where one parent carried the green cotyledon mutation). F1 seeds generated by crossing JI 2110 (*ii*, green cotyledon) with cv. Cameor (*II*, wild-type yellow cotyledon) were expected to show a complemented (*Ii*, yellow cotyledon) phenotype and, where one parent showed a compound starch grain morphology (JI 1194 or JI 1208, Figure 4), complementation by JI 2110 was expected to yield simple starch grains. The *rug 5* mutant was not included in allelism tests, since mutations in this gene lead to compound starch grains [16,20], which are not evident in JI 2110.

Initial crosses gave the surprising result that the seed shape phenotype of the F1 obtained from reciprocal crosses differed. Where JI 2110 was the pollen donor, the phenotype of the F1 seeds showed complementation of the mutations at *rug 3*, *rug 4* (Figure 4A) and, as expected from the genotyping results, at *r* and *rb* loci (not shown). However, when JI 2110 was the maternal parent of crosses, the F1 seeds showed a wrinkled appearance (Figure 4A). Overall, the results suggested that the mutation in JI 2110 affects the testa of the seeds, which is maternal tissue, and that this impacts ultimately on seed shape. In contrast, the mutations at *r*, *rb* and the induced mutations used in test crosses all impact on cotyledonary metabolism, and are dependent on the genotype of the embryo (including the cotyledons) and independent of the maternal (including testa) genotype. As the mutations are recessive, those affecting the embryo are not reflected in the phenotype of the heterozygous seeds generated. The starch grain phenotypes of *r*, *rb* and JI 2110 are shown in Figure 4B.

The genetics of the wrinkled-seeded trait in JI 2110 were investigated further by generating a set of recombinant inbred lines (RILs) from a cross with the round-seeded (*RR*) genotype, JI 281, developing a genetic map and carrying out phenotypic analysis of the RILs.

### 2.4. Genetic Mapping of the JI 2110 Seed Phenotype

**Figure 5:**
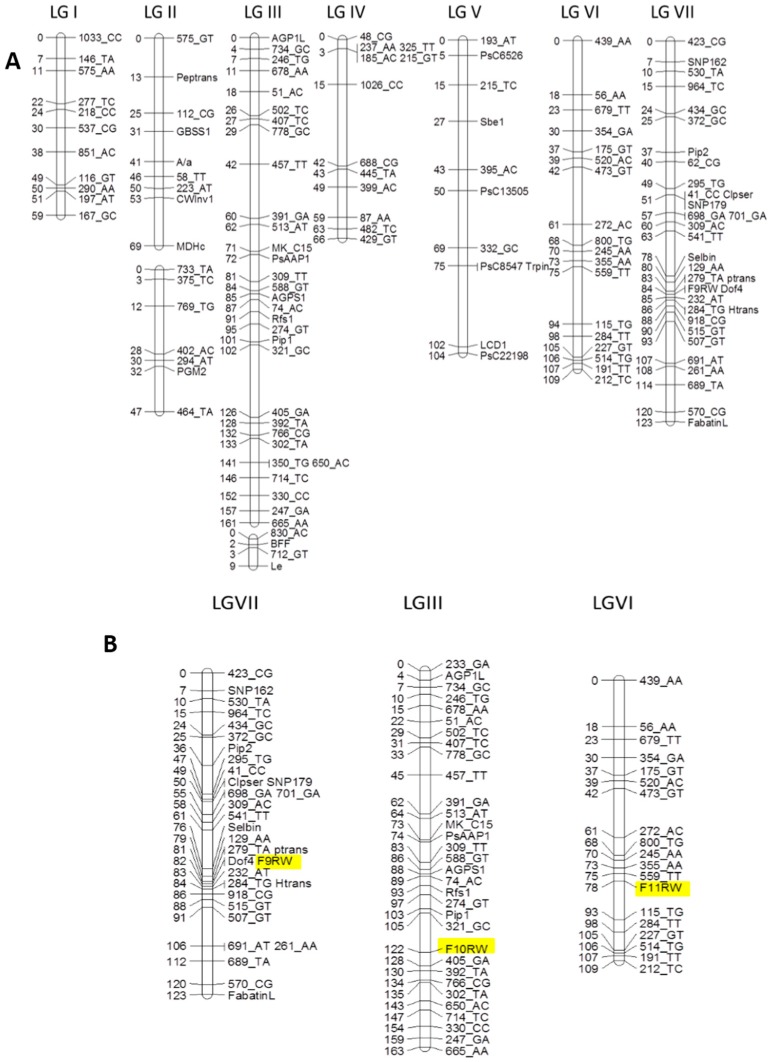
(**A**) Genetic linkage map developed for JI 281 × JI 2110 RILs, based on SSAP, gene-specific and morphological markers; (**B**) The major genetic positions determined for the seed phenotype variation (highlighted) in JI 281 × JI 2110, based on individual generation scores. The major map positions determined for seed shape phenotype over three generations (F9, F10 and F11, round, R, wrinkled, W) are highlighted in yellow.

In order to map the variant testa phenotype in JI 2110, reciprocal crosses between JI 281 and JI 2110 were used to generate a set of 205 RILs, for which a genetic map was constructed. Reciprocal crosses were made to facilitate investigations of maternal effects. No losses of parent plants or RILs occurred due to germination or other growth problems. DNA was extracted from RILs at F8 and 202 RILs scored for polymorphisms/variation associated with 103 sequence-specific amplified polymorphic (SSAP) [21], 28 gene-specific and 2 phenotypic (*a*, controlling anthocyanin production; *le*, plant height) markers, from which a map was assembled using the JoinMap^®^ programme (Kyazma, Wageningen, The Netherlands) (Figure 5 and Table S1). JI 281 was chosen as a rich source of polymorphic markers and SSAP marker analysis alone was sufficient to generate a genetic map based on six of the seven linkage groups (LG) expected for pea [22,23]. Additional markers (gene-specific) were required to coalesce LG V into a single group. Some markers specific to LG III (linked to *le*) remained unlinked (Figure 5).

Phenotypic analysis of seed shape was carried out for F9, F10, and F11 seed batches, when seeds were fully mature and using three seeds from the middle of a pod from a number of pods to visually score round- or wrinkled-seeded phenotypes. Although the irregular seed shape phenotype of JI 2110 and a subset of the RILs was noticeably more or less pronounced at different generations grown in different environmental conditions, it became clear that the phenotype was not segregating in the 1:1 ratio expected for advanced generation RILs, which suggested that more than one genetic locus was responsible.

The seed phenotype scores for the RILs over three generations (F9-F11) were tabulated according to those that were in agreement over all (“agreed”, 3/3) or majority (“majority”, 2/3) scores, and separately those fewer lines for which an extreme (round- or wrinkled-seeded) phenotype (‘Extreme’) was scored.

A 1:1 segregation ratio is expected for the segregation of the alleles of a single gene in a RI population. The “majority” and “agreed” scores were not consistent with this interpretation (Table 3). A 3:1 segregation ratio is expected for the segregation of the alleles of two unlinked genes in a RIL population. The “majority” and “agreed” scores were consistent with this interpretation with *p* values of 0.4–0.8 (Table 3). The “extreme” scores could be consistent with either a 1:1 or a 3:1 segregation ratio (the *p* value is 0.02 for the latter but much higher for the former, Table 3). Taken together, these data are consistent with a two locus interpretation for the control of this trait. In contrast, the ratios for plant height (*Le/le*) showed a good fit to 1:1 as expected (*p* > 0.5 for F8 plants).

Associating the seed phenotype scores with the genetic marker data identified two genetic loci at F10 and F11 generations (LG III and LG VI) and an additional locus at the F9 generation (LG VII, Figure 5B). Analysis of the “extreme” scores alone showed association with the LG VI locus (near 520_AC). These regions of the genetic map were targeted for addition of further genetic markers, using gene-specific polymorphisms [24,25]. The gene encoding fabatin mapped close to the LG VII locus, but this was detected at one generation only. Analysis of the common seed phenotypes scored at three generations (F9, F10 and F11) showed association to *AgpS1*, *Rfs*, and *AAP1* on LG III, encoding the small subunit 1 of ADP-glucose pyrophosphorylase, raffinose synthase, and amino acid transporter 1, respectively. Sequence analysis of these genes in JI 281 and JI 2110 revealed single nucleotide polymorphisms which facilitated mapping but no unique difference between JI 2110 and other (JI 281 and database) sequences for the *AgpS1* (X96764) and *Rfs* (AJ426475) genes. The amino acid sequences for AgpS1 in the two genotypes were identical, likely precluding a direct involvement of these genes in determining phenotype. Sequence analysis of AAP1 in the two parents predicted proteins that differ by four residues but with only one residue difference between JI 2110 and the database accession AAX56951.1; none of the changes are predicted (available online: http://blocks.fhcrc.org/proweb/input/) to impact on AAP1 function. The protein sequences predicted for these genes are presented in Figure S2.

The combined data suggested that more than one genetic locus control the maternally determined seed trait in JI 2110, and that one of the loci involved is likely to reside on LG III. To facilitate the identification of candidate genes in the genetic map regions affected, metabolite analysis of parent seeds was undertaken to examine the hypothesis that the testa of JI 2110 may show biochemical differences analogous to those reported for cotyledons of *r*, *rb*, and other *rugosus* mutations [2], impacting on the profile of sucrose-derived metabolites.

### 2.5. Compositional and Metabolite Analysis of Cotyledons and Testas in JI 2110

**Figure 6:**
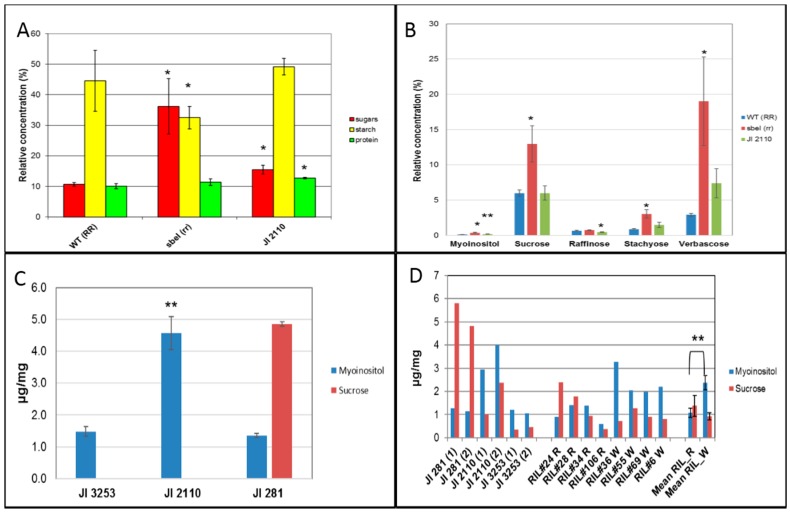
(**A**) Relative concentrations (% mg dry weight) of total soluble sugars, starch, and protein in cotyledons of JI 2110 in comparison with near-isogenic wild-type (WT, *RR*) and wrinkled-seeded *sbeI* (*rr*) mutant lines. Sugars were measured relative to an internal standard, and protein relative to a standard. Bars indicate standard deviation; three replicate seeds per line. * *p* < 0.05, determined by *t*-test; (**B**) Relative concentrations (% mg dry weight) of myoinositol, sucrose, raffinose, stachyose, and verbascose in cotyledons of JI 2110 in comparison with near-isogenic wild-type (WT, *RR*) and wrinkled-seeded *sbeI* (*rr*) mutant lines. Bars indicate standard deviation; three replicate seeds per line. * *p* < 0.05, ** *p* < 0.01, determined by *t*-test; (**C**) Concentrations of myoinositol and sucrose (μg/mg dry weight) in testas of JI 2110 in comparison with testas of the wild-type (round-seeded) genotypes, JI 281 and JI 3253. Bars indicate standard deviation; three replicate batches of testas per line. Standards of each compound enabled detection and quantification; metabolites not shown were below the lowest calibration points of the standards. ** *p* < 0.01, determined by *t*-test. ((**A**–**C**) *, ** denote comparisons of JI 2110 or *sbeI* with wild-type lines); (**D**) Concentrations of myoinositol and sucrose (μg/mg dry weight, determined using standards of each compound) in testas of JI 2110, JI 281, JI 3253 (1, 2, two replicate seed batches in every case) and a set of white-flowered JI 281 × JI 2110 RILs (four round-seeded, R; four wrinkled-seeded, W). Mean values for the R and W RILs are shown with standard error bars. ** *p* = 0.01, determined by *t*-test of myoinositol concentration in R and W RILs.

The relative concentrations of soluble sugars, starch, and protein in JI 2110 cotyledons showed greater similarity to those of a wild-type round-seeded (*RR*) line than to those of its near-isogenic wrinkled-seeded (*rr*) counterpart (Figure 6A). The concentration of starch in JI 2110 was not significantly different to that of the wild type, whereas small increases in total sugar and protein concentrations were observed in the former (*p* < 0.05, Figure 6A). The concentrations of starch and soluble sugars were significantly lower and higher, respectively (*p* < 0.05), in the *sbeI* (*r*) wrinkled-seeded mutant compared with its near-isogenic wild type, as previously reported [2,6]. Analysis of individual soluble sugars in the cotyledons of JI 2110 again showed a profile that was more similar to wild type than to the wrinkled-seeded *r* genotype (Figure 6B). In the *sbeI* mutant, relative concentrations of sucrose, stachyose, and verbascose were higher than in the wild type (*p* < 0.05). Although JI 2110 showed a higher concentration of verbascose than that in the wild-type line, the difference was not significant (*p* = 0.06). JI 2110 showed a lower concentration of raffinose (*p* < 0.05) but higher myoinositol (*p* < 0.01), when compared with the wild-type line (Figure 6B).

Analysis of the same range of soluble sugars and galactinol in the testas of JI 2110 and the round-seeded genotypes, JI 281(second parent of the RILs) and JI 3253 (cv. Cameor), revealed patterns that were distinct for every line (Figure 6C) and differed from the cotyledon profiles. Four metabolites overall (galactinol, raffinose, stachyose, verbascose) and, for two lines, sucrose fell below the measurement limit in this experiment, where standards of every compound were used to determine absolute concentrations. Sucrose was the most abundant metabolite measured in JI 281, whereas myoinositol predominated in JI 3253 and in JI 2110, with much higher concentrations (*p* < 0.01) in JI 2110 than in either of the other two genotypes (Figure 6C).

Analysis of testas from a second batch of seeds of these three genotypes confirmed the high sucrose concentrations in JI 281 and the higher myoinositol concentrations in JI 2110 (Figure 6D); in JI 2110 and JI 3253 testas, sucrose peaks fell at or just below the measurement limit. The testa of JI 281 is coloured and thicker than that of the other two genotypes, reflecting the dominant allele at the *A* locus, controlling anthocyanin production. Analysis of testas from a selection of JI 281 × JI 2110 RILs which had been scored consistently as round- or wrinkled-seeded (all white-flowered, lacking anthocyanins) showed that the wrinkled-seeded RILs contained higher concentrations of myoinositol (Figure 6D, *p* = 0.01), with a mean value that was more than two times higher than that of the round-seeded RILs, and in agreement with differences seen between the parents. There was no difference in sucrose concentrations between the two set of RILs (*p* = 0.39). It may be that the higher sucrose concentrations in the JI 281 testas reflect the status of the allele at the *A* locus, since all the white-flowered RILs analysed showed relatively low sucrose concentrations in their testas (Figure 6D). The difference in myoinositol concentration between the two groups of RILs (Figure 6D), and the higher concentration overall of this metabolite in JI 2110 may provide the basis for explaining the contrasting wrinkled-seeded phenotype described here, which is controlled maternally.

## 3. Discussion

In this work, we describe a novel type of wrinkled seed present in a pea germplasm collection, following high-throughput genotyping by multiplex PCR assays designed to amplify different portions of wild-type and mutant *SbeI*/*sbeI* alleles. The majority of wrinkled-seeded lines were classified as having the *sbeI* insertion, described previously as the mutation underlying the *r* locus [1]. All of the wrinkled-seeded types associated with the *r* locus carried the insertion allele. Since the *rb* mutation was not known in Europe prior to the 1930s [11,12], the *sbeI* analysis provided here supports the claim that Mendel's wrinkled-seeded allele was that described by Bhattacharyya et al. [1]. The sequence of the insertion element provided here (Figure 1A) facilitates genomic screens based on the insertion itself, but the most robust assays of germplasm avoid primers based on the insertion due to the repetitive nature of its sequence, which appears to diminish the reliability of priming and/or the fidelity of polymerases. Here, we provide the conditions for two types of basic PCR assays, designed to distinguish *RR*/*Rr* from *rr* genotypes (Figure 2), which should have broad utility from education to breeding programmes. A predicted consequence of the insertion for SbeI is that the carboxy-terminal 62 amino acids are missing from the mutant protein, with 30 additional amino acids predicted by read-through within the insertion (Figure 1); in contrast, Bhattacharyya et al. [1] merely suggest a loss of the last 61 amino acids of the wild-type protein.

Following *sbeI* genotyping and phenotyping, 15 lines were identified which were wrinkled-seeded, but not *r* mutants (Table 2). Thirteen of these were determined to be *rb* mutants, following identification of a mutation in the gene encoding the large subunit of ADP-glucose pyrophosphorylase. The mutation described here, common to all *rb* lines studied, is a deletion of nine base pairs that leads to a loss of three amino acids (ATP) from the mature protein (Figure 3A), and is likely to provide an explanation for the changes to the catalytic properties of the mutant enzyme described previously [10]. The region of the protein affected includes residues which interact with ADP-Glc and ATP [26] and the three missing amino acids may contribute directly to these interactions, rather than simply through determining structural conformation in this region of the protein. As with the *r* mutation above, knowledge of this deletion will enable screening programmes, particularly where mutations are combined and scoring by phenotype is highly unreliable.

Two wrinkled-seeded germplasm lines were shown to be neither *r* nor *rb* mutants. One of these (JI 2110) was chosen for detailed study. The phenotype of this line was observed to be distinct in many respects from that of wrinkled-seeded mutants described earlier for pea. The main distinction was the maternal determination of the phenotype, such that F1 seeds borne on JI 2110 consistently had a wrinkled-seeded phenotype (Figure 4A). This was in sharp contrast to the wrinkled-seeded mutants described previously, where the zygote genotype influences seed shape. Analysis of RILs derived by crossing JI 2110 with the round-seeded JI 281 indicated strongly that more than one locus controlled seed shape in JI 2110. This is also supported by the fact that mutagenesis in pea has not delivered a mutant line such as JI 2110 [2,5], where many alleles of five distinct genes influencing seed shape were identified. Equally, TILLING populations of pea [27] have led to the identification of wrinkled-seeded lines, but none of these shows features similar to JI 2110 (unpublished data; available online: http://urgv.evry.inra.fr/UTILLdb). On the other hand, there are additional genes for which mutations might be expected to lead to changes in seed shape in pea and which have not been described. One of these is a transcription factor, WRINKLED1, described in Arabidopsis as controlling changes in seed composition, chiefly fatty acid metabolism and lipid concentrations [28,29,30].

Reciprocal crosses between JI 2110 and JI 281 were made to investigate whether there was maternal inheritance in addition to maternal determination. In the RIL population, there was no evidence for maternal inheritance, but this does not rule out maternal inheritance of a determinant active when the cotyledon and testa are of different genotypes. The strong environmental effect on the seed phenotype of JI 2110 led to difficulties in obtaining consistent scores across generations. Nevertheless, the consistently agreed and majority scores across three generations led to the identification of genetic loci associated with the trait. Of the two loci expected from segregation ratios and their fit to a 3:1 ratio (Table 3), linkage group III may provide the best lead for the identification of candidate genes. It is worth noting that, despite the fit to a 3:1 ratio, the segregation could also be compatible with more than two genetic loci for which epistatic interactions are not simple. Investigations of some genes on LG III associated with the JI 2110 trait did not reveal any substantive difference between the two parents. Included here was *AgpS1*, one of the small subunits of ADP-glucose pyrophosphorylase. Although this gene might provide a strong candidature for a wrinkled-seeded trait, analogous to *rb*, neither natural nor mutagen-induced wrinkled-seeded mutants have been described for this gene in pea. This may be explained readily by the fact that there are two small subunit genes (*AgpS1* and *AgpS2*) for this enzyme, and downregulation of both by RNAi is the likely explanation for the alterations to seed composition and seed shape reported in the study of Weigelt et al. [31]. In the studies of the JI 2110 phenotype reported here, an association with the genetic location of the *AgpS2* gene (LG I) was not observed, making it unlikely that the JI 2110 phenotype could be explained by changes to the two *AgpS* genes.

The biochemical differences measured in JI 2110 and wrinkled-seeded RILs, compared with round-seeded counterparts, indicated that myoinositol metabolism is altered in JI 2110. Preliminary analysis of the second line, JI 1417, identified as being neither *r* nor *rb*, indicated that its phenotype behaves in a similar way to that of JI 2110 in crosses (data not shown) but we cannot conclude that the genetic basis for the trait is the same in both lines. Further work should examine the expression of genes within the pathway leading to and from myoinositol, together with quantitative analysis of the associated metabolites, in JI 2110. It seems reasonable to hypothesise that a mutation leading to a higher accumulation of myoinositol in testas could lead to a higher osmotic pressure within cells and wrinkling of the testa, analogous to the effects of sucrose accumulation in the *rugosus* mutants. The RILs developed here should facilitate such analyses of the testa trait in JI 2110. Myoinositol metabolism is linked to that of phytate, but analysis of low phytate mutants in pea revealed no differences in a gene encoding myoinositol phosphate synthase (MIPS) between wild-type and mutant genotypes [32]. There appears to be two *MIPS* genes in pea, based on the pea gene atlas [33], with one likely to be on the pea LG III, based on synteny with *Medicago truncatula* chromosome 3 [34]. In contrast, a locus controlling phytate concentration has been mapped to LG V [35], with phytate concentration being inherited simply [36]. Studies of MIPS in Arabidopsis have reported that seeds of *mips* mutants generate a significant percentage of wrinkled seeds [37], providing support for investigations of these genes in pea, with a focus on LG III candidates. Although the seed cotyledon composition of JI 2110 was more similar to that of a round-seeded than that of a wrinkled-seeded (*rr*) genotype, the small increase in total sugars, coupled with a higher myoinositol concentration in testas as well as in cotyledons of JI 2110 (Figure 6), may prove to be of interest to breeding programmes targeting sweetness in food products. In seeds, high sucrose concentrations can lead to a trade-off with germination traits [38], and JI 2110 may offer some compromise in this regard.

The present work highlights the value of combined phenotyping and high-throughput genotyping to identify novel variants in germplasm collections. Equally, the work highlighted the deficiencies of both, in that genotyping has an associated failure rate which needs to be minimised through the design of robust assays. The problem associated with heterozygous lines and scores was addressed in this work, and these lines were low in number. They could be more widespread, however, and evade detection if, for example, a line was hemizygous for a deletion allele. Although test lines can be used to increase robustness of assays, germplasm accessions are by their nature diverse and divergence in primer sites leads to problems. In the work reported here, the numbers of such failures were relatively low. Difficulties with phenotype data also needed to be addressed. The cases where a lack of agreement was found when genotypes were re-phenotyped served to highlight the fact that, for accurate phenotyping of seeds, care must be taken to ensure that seeds have matured properly on plants. Premature harvest of round-seeded genotypes will lead to a wrinkled appearance of the seeds, which lose residual water more rapidly than would be the norm.

It is clear that Mendel [4] considered this last point very carefully in his work (“It is almost superfluous to mention that the pods must remain on the plants until they are thoroughly ripened and have become dried, since it is only then that the shape and colour of the seed are fully developed”). Nonetheless, even with such careful management of JI 281 × JI 2110 RILs, their phenotypes proved difficult to score visually and, if Mendel had chosen JI 2110 for his experiments, he may well have chosen not to investigate the seed shape trait in pea. Mendel made the point that, for his analysis, characters should differ “constantly” and those which “do not permit of a sharp and certain separation... of a “more or less” nature ... could not be utilized”. He did however note that traits which do not follow simple and discrete segregation and show such "enigmatical results, however, might probably be explained by the law governing [*Pisum*] if ... a combination of two or more entirely independent [characters]... individually act like any other constant character". In contrast Galton [39], who also studied the segregation of seed shape in pea, found the “misshape and corrugations” problematic for his analysis of seed diameters and so used seed weight as a surrogate. Having been presented with discrete segregation, Galton chose to ignore it, whereas Mendel when presented with continuous variation considered it likely to be due to the interaction of multiple genes.

Besides the interest in this work from breeders seeking to broaden the genetic base utilised in their vegetable breeding programmes and to screen for known mutations with confidence, a greater interest in genetic diversity is being driven by research on the health benefits provided by variant plant starches in preventing Type II diabetes [9]. The work described here will also inform these studies.

## 4. Materials and Methods

### 4.1. Plant Materials and Germplasm Genotype Screens

The pea germplasm collection curated by the John Innes Centre Germplasm Resources Unit (available online: http://www.jic.ac.uk/germplasm/) was used throughout this study. Near-isogenic pea lines (BC1/19RR, BC1/19rr) with wild-type *SbeI* (*RR*) and mutant *sbeI* (*rr*) alleles, respectively, were also provided by the unit. A mapping population was produced by reciprocal crosses between JI 2110 with JI 281 and recombinant inbred lines (RILs) developed to F11 by single seed descent from F2 lines; the first 103 RILs were from JI 281 × JI 2110 and the remaining 102 RILs were from JI 2110 × JI 281.

The germplasm screen was based on ~2800 *Pisum* accessions, representing the broad genetic diversity available across the JIC *Pisum* accessions (File S1). Printed DNA plates containing DNA from individual plants [13] were used for high-throughput genetic screening as described previously [15]. Multiplex PCR assays were designed to yield amplicons of 50–300 bp, based on predicted discrete gene-specific products which could be identified both by fluorescent label and size [40]. Separation of fluorescently labelled amplicons was carried out on an ABI3730 instrument (Life Technologies, Carlsbad, CA, USA). One primer of every pair carried a fluorescently labelled tag detector probe (either Fam, blue, or Vic, green), to facilitate the detection of products, and additional bases (T) added to primers as necessary to distinguish gene products of otherwise similar predicted sizes (see image in File S1).

The screens for genetic variation in the pea starch-branching gene, *sbeI*, were based on the sequence of NCBI accession X80009, determined for a wild-type round-seeded line (BC1/9RR, derived from introgression of the wild-type allele into the wrinkled-seeded JI 430) and from sequence determined for the mutant allele in JI 1194 (Figure 1). Gene primers for *sbeI* are listed in Table 4 and their positions indicated in Figure 1.

The primers Ps-SBEI-F4 and Ps-SBEI-R4 were used to develop a facile PCR diagnostic test for the *sbeI* mutation, with or without a third primer (*r*-element-F2) designed for a triplex assay. The remaining primers were used in the high-throughput germplasm screen, with modifications to the primers as indicated in red font (product length extension, fluorescent label). The colour of the products expected from the primer combinations used is indicated.

For routine screening of *sbeI* insertion mutants, the primers Ps-SBEI-F4 and Ps-SBEI-R4 provided a diagnostic assay, based on amplification by PCR of the wild-type allele lacking the insertion from the mutant allele containing the insertion. The assay conditions proved critical to the amplification of the mutant allele. Briefly, conditions were as follows: 20 µL reactions contained 200 ng DNA, 0.75 mM total dNTP, 0.25 µM each of the Ps-SBEI-F4 and Ps-SBEI-R4 primers and 1 unit of TaKaRa Ex Taq^®^ DNA polymerase (TaKaRa, Seoul, Korea), using the buffer (containing 20 mM MgCl_2_) and dNTP solutions provided by the manufacturer. PCR conditions were: 95 °C for 2 min; followed by: 94 °C for 30 s, 65 °C for 45 s, 72 °C for 90 s, repeated 34 times; 72 °C for 5 min, 10 °C to end. The triplex assay designed to assay wild-type and mutant alleles, based on amplicons of similar size, utilised 20 µL reactions as for the simplex reaction, except that a third primer (r-element-F2, 0.25 µM final concentration) and 0.5 unit of the polymerase was used per reaction. Touch-down PCR conditions (65–55°C) for this assay were: 98 °C for 60 s, followed by: 98 °C for 10 s, 65 °C (−1 °C/cycle) for 30 s, 72 °C for 30 s, repeated nine times; 98 °C for 10 s, 55 °C for 30 s, 72 °C for 30 s, repeated 25 times; 72 °C for 5 min, 15 °C for 5 min. In the triplex reaction, the third primer and the shorter extension time mean that the larger product amplified in the *sbeI-ins* mutant by Ps-SBEI-F4 and Ps-SBEI-R4 primers alone is not seen (Figure 2).

Further screening of germplasm variants was carried out by standard PCR, based on determination of the sequence for the mutant allele of the gene encoding the large subunit of ADP-glucose pyrophosphorylase at the *rb* locus (Figure S1 with primers listed). The mutant *Agpl* sequence has been deposited in GenBank (accession number MF186819).

### 4.2. Allelism Tests of Seed Phenotype

The novel wrinkled-seeded variant, JI 2110, was crossed with lines carrying mutations at *r*, *rb*, *rug 3*, and *rug 4* genetic loci. Crosses were checked for heterozygosity by the phenotype of F1 seeds, their cotyledon colour and, where necessary, by checking (gel analysis or sequencing) regions of genes that showed polymorphism between the two parents. The appearance of the starch granule provided a useful trait, following non-destructive drilling of a small portion of seed meal from individual F1 seeds. Meal samples were placed on microscope slides with water and starch grain morphology was observed by light microscopy, before and after staining with iodine.

### 4.3. Genetic Mapping of Novel Variant Seed Phenotype

Reciprocal crosses were established between JI 2110 and JI 281, the latter genotype being a proven source of polymorphic markers. A set of recombinant inbred lines was generated by single seed descent to F11. A genetic map was constructed using genomic DNA from lines at F8 and fluorescently-labelled SSAP markers based on the PDR1 element [21], plus a set of gene-specific markers designed to increase marker density in certain regions of the map. Gene-specific markers were developed, based on genetic synteny between pea and *Medicago truncatula*, and the genetic maps available [24,25]. A total of 103 SSAP markers and 28 gene-specific markers (Table S1) have been used to generate the JI 281 × JI 2110 genetic map, in addition to two phenotypic markers (*le* and *a*). Maps were assembled using JoinMap^®^ 3.0 (Kyazma, Wageningen, The Netherlands). The seed shape phenotype was scored independently in three generations (F9, F10 and F11), and logged according to agreement (”agreed”, where the score was the same in all generations), a majority score (“majority”, where the accepted score was the same in two of the three generations), and extreme (“extreme”, where the phenotype was notably clear and similar to the difference between the parents).

### 4.4. Metabolite Analysis of Cotyledon and Testa Samples

Cotyledon meal samples were extracted and analysed for their soluble sugars by GC/MS as described previously, using phenyl-β-d-glucopyranoside as an internal standard, against which peak areas were compared to give relative concentrations of sucrose and sucrose-derived oligosaccharides [41]. Starch concentrations were determined as described [42]. Protein concentrations were determined relative to bovine serum albumin, using the Bradford assay [43].

Samples of testas were prepared by weighing accurately 5 mg of freeze dried material into a 10 mL grade A volumetric flask, adding 50% ethanol containing 50 mg/L melezitose as an internal standard, and sonicating for 15 min at 20 °C. The samples were centrifuged to provide supernatants for analysis. Standard solutions of galactinol (sc-228240, Insight Biotechnology Ltd., Wembley, UK), myoinositol (Sigma 87-89-8, St. Louis, MI, USA), raffinose (Sigma 17629-30-0), stachyose (Sigma 10094-58-3), verbascose (Megazyme O-VER, Chicago, IL, USA) , and sucrose (Sigma 57-50-1) were prepared to provide calibration curves for determination of the concentrations of the compounds in test samples.

Testa samples and standards were analysed by liquid chromatography-mass spectrometry (LC/MS), using a Shimadzu system comprising NexeraX2 LC-30AD binary pumps degassers, SIL-30AC autosampler, Prominence CTO-20AC column oven, and a LCMS2020 single quadrupole mass spectrometer (Shimadzu UK Ltd., Milton Keynes, UK). Chromatographic separation was achieved using an Accucore-150-Amide-HILIC column (100 × 2.1 mm × 2.6 µm, Thermo Scientific, Waltham, MA, USA), maintained at 25 °C. The mobile phase consisted of 0.1% formic acid (A) and acetonitrile (B). Initial composition was 80% B changing to 60% B over 8 min, holding for 2 min before returning to 80% B over 0.5 min, and equilibration for a further 4.5 min. The flow rate was 400 µL/min. MS detection was achieved using negative ionisation with a desolvation line temperature of 250 °C, heat block temperature of 200 °C, nebulising gas flow of 1.5 L/min, and drying gas flow of 15 L/min, monitoring ions at *m*/*z* 224.9, 386.9, 549.0, 711.0, 873.0.

## 5. Conclusions

The work described here identifies a novel source of genetic variation controlling wrinkled-seeded phenotypes in pea germplasm, in addition to providing new insights into the previously characterized *r* and *rb* mutations. The novel wrinkled-seeded phenotype identified in JI 2110 is maternally determined and is likely to be controlled by two genetic loci. Metabolite profiling revealed changes in myoinositol concentration in JI 2110, particularly in the testa, suggesting an explanation for the seed phenotype.

## Figures and Tables

**Table 1:** Summary data obtained from genetic analysis of the presence or absence of the *sbeI* insertion mutation in a pea germplasm resource for which phenotypic data were available. The numbers of accessions scored as wild type (*SbeI-wt*), mutant (*sbeI-ins*) or heterozygous (*SbeI-wt* and *sbeI-ins*) among the different phenotype classes are listed.

Phenotypic Description	Marker Assay Allelic Classes			Total
	*SbeI-wt*	*sbeI-ins*	*SbeI-wt & sbeI-ins* (Presumed Heterozygotes)	
Round/Dimpled	1766	42	6	1814
Marrowfat	168	29	2	199
Wrinkled	59	716	4	779
Total	1993	787	12	2792

**Table 2:** The identities of 15 germplasm accessions, lacking the *sbeI* insertion mutation, of which 13 are *rb* and two (JI 2110, JI 1417) are neither *r* or *rb*. WT, wild type *AgpL* score, where the deletion is absent (−); the deletion was present in lines shown with a + score.

Wrinkled-Seeded Phenotype (*Sbei* Insertion Minus)	*AgpL* Genotype Score	
	*rb* Deletion	WT
JI 399	+	−
JI 492	+	−
JI 528	+	−
JI 601	+	−
JI 1156	+	−
JI 1157	+	−
JI 1408	+	−
JI 2012	+	−
JI 2107	+	−
JI 2109	+	−
JI 2111	+	−
JI 2368	+	−
JI 2369	+	−
JI 1417	−	+
JI 2110	−	+

**Table 3:** Analysis of seed phenotype segregation in JI 281 × JI 2110 RILs (F9, F10 and F11), with chi square and probability values for a goodness of fit to 3:1 and 1:1 segregation ratios. Scores are classified according to their agreement across three generations (F9, F10 and F11) for round-seeded (A class) or wrinkled-seeded (B class) phenotype.

Observed	Class Group			Chi Square Analysis			
	A	B	total				
Agreed scores	78	21	99				
Majority scores	109	38	147				
Extreme scores	17	13	30				
				A	B		
Expected (3:1)				Chi Square	Chi Square	total	*p*
Agreed scores	74.25	24.75		0.19	0.57	0.76	0.3841
Majority scores	110.25	36.75		0.01	0.04	0.06	0.8118
Extreme scores	22.5	7.5		1.34	4.03	5.38	0.0204
Expected (1:1)				Chi Square	Chi Square	total	*p*
Agreed scores	49.5	49.5		16.41	16.41	32.82	0.0000
Majority scores	73.5	73.5		17.15	17.15	34.29	0.0000
Extreme scores	15	15		0.27	0.27	0.53	0.4652

**Table 4:** Primers used in screening for *sbeI* variants in pea germplasm. The direction of the primer read is indicated: F, forward; R, reverse.

Primer Name	Sequence (5′–3′)	Direction	Colour
Ps-SBEI-F4	GAACAGCAGTGGTGTATGCC	F	-
Ps-SBEI-R4	GACTTGATTGCACTGATGCC	R	-
r-element-F2	ATGGATCAAATTGCCACCC	F	-
Sbe1F4F	Fam-GATCATTCCTCCACCCGGTA	F	Blue
Sbe1R4e	TTTTTGCTGACGGTGAGCTTGC	R	Blue
Sbe1rcFV	Vic-CCTTGCGGATGGAGTTGAA	F	Green
Sbe1rIR	TCCATTTTGCCACCCCTAATT	R	Green
Sbe1rDR	AACTCCAACTGACAGCTCAATTGG	R	Green

## Supplementary Materials

Supplementary materials can be found at www.mdpi.com/1422-0067/18/6/1205/s1.
