# Supplementary material for: Genetic Variation Controlling Wrinkled Seed Phenotypes in Pisum: How Lucky Was Mendel?
Source: Int J Mol Sci. 2017 Jun 6;18(6):1205. doi: 10.3390/ijms18061205 (PMC5486028; doi:10.3390/ijms18061205)
Supplement: Supplementary file 1 [file ijms-18-01205-s001.zip › ijms-196391-supplementary/ijms-196391-supplefigures-0607.pdf]

Int. J. Mol. Sci. **2017**, *18*, xx

Genetic Variation Controlling Wrinkled Seed Phenotypes in *Pisum*: How Lucky Was Mendel?

Tracey Rayner [1,*], Carol Moreau1 *, Mike Ambrose [1], Peter G. Isaac [2], Noel Ellis [3] and Claire Domoney1 *

AAATATAAATTCTAATATGATGCTACCATGATTTATGAAACTCAATCAACCTATCCTATAATCATTAATGTTGTTTACAAATTTTGCAACAT
GACAAATGACTTCCACATACATCCAAACCCCGCCATCAAATGTCAGATGTAAGCATCCTTAAATCATGAACCAGTAATTTTTTGTTACATAA
GATAAAACTACAAATACTTCCCTTTTAAACAACACTTTATTTTTGACTGAAAGTTCTAACACTTTGTTACATTTTTACAACACATTGAAGCA
AATAACAACCGCAAAAGTTCACATCAAGTGAAAATGACATTGTCTTTGTTGTGTAATGAGAATCCCATTACAACCTTGGACTTTTGTGAGTG
AATCATTTCATCAACCATGTTTCCAAAGGCAAACAAATATCAAAAAATATAATAATATTTTTTTTAGAAATTGAGGTTTCTGATTTCTCTCA
ATAAAGGCAACACCTTTTCCTTCATCACTACTCAATAATTAGTTTTAGATTCACATTCTTCTCTTTGTTTCTCTAGATTCTATATTTTAGTT
GTGACCTTTCACTACTAGCATTGTTTCTCTCTTATTCTCTTGGTCTGAGTTTGAACAAACTCAAAAAAAAAGCTTAGTTTTTTGAGGTTACT
ACAATGGCTTCTGGTTGTGTGAGCTTGAAAACCAACACCCATTTTCCAAATTCTAAAAAAGGTTCTTTTTTTGGGGAAAGAATCAAAGGAAG
CTTGAAAAACAGTTCATGGGTCACTACCCAGAAGAAGATCAAACCTGCTTCTTTTTCTGCTATTCTTACTTCAGATGACCCCAAAGGTTCCC
TGGTAAACTCAGTTTCATTCTGGGTTTCACTTTTTGCTTCCAATTCTGAAAAAAAGAAAGACTTTTTTTTTCTCCCATTATATGACATAACT
TTTTTATGTTAATTATTTTGCTACATTTTGTTTGGTATATGATTATGATTATGATTATGATTTTGAGTGTATGTTTTGAAATTCAGAATTTG
CAAGTGCCTTCATTTCTGAGACTAAGAGCTGATCCAAAAAATGTGATTTCCATTGTGTTGGGAGGAGGGCCTGGAACACATCTCTATCCTCT
TACCAAACGAGCTGCGGTGAGTGAGTTTTATGATATGAATTGTTGTCTGAATTCTAACATTTTGGTGTTGTTTGTAACTTTGTGCTGTTTGT
GATGAAGATTTGTTTGCTGTGTTTGTTTTGAAGGTTCCTGTTGGAGGATGCTATAGGCTTATAGACATTCCAATGAGCAACTGCATCAATAG
TGGCATCAACAAGATATTTGTGCTGACTCAGTTCAACTCTGCTTCACTAAATCGTCACATCGCTCGCACCTATTTCGGAAATGGTGTCAACT
TTGGAGATGGATTTGTGGAGGTAATCATGCATCACCATCATGAAAATGGATGGAAATTAGTACTTTTTTTTTCTCGCATTTTTTATATTGGA
ACAATCTTTTAGCATACTTTGTTTATCTTTTGGTCGAACTCCGAATAACTAGGATCCGTCTTTTCTGGGATTTAGAGAGTTAAGAACAAAAA
AATATATTTTCATGCAATGAGTTTTGAGATGAACATGACAGTTGCCAACAAACAAAACATAAGTAATTCGGTTTGCGTAGAGAAATACCGAT
CCAGTGAAGGAGATTCTTGTTGATGAATTTCAAGTTTGTTTCTATTTATGATTCATGAGGACTAATTAAAGTCAATCTAAATGTCAAAATAG
GTTCTGGCGGCGACACAAACACCAGGAGAAGCTGGGAAGAAGTGGTTTCAAGGAACTGCAGATGCTGTGAGACAATTTACCTGGATATTTGA
GGTAGACAAAGGATTTTTGTTGTTGTTGTATATACATTTCGATAAATAATAGATTCTTTTGTTCTCATTTTTGAGCTTGTCAATAAGTAATA
GATTGTTTGTGGTAGGATGCCAAGAATATAAACGTCGAGAATGTATTGATCTTGGCCGGAGATCATTTATATCGAATGGATTACATGGATCT
ATTGCAGGTATATTGTGAATGTTTTGTAGAGTGGATTGTTTTTCATTTCATGTTCTAGAGTTTTCTGATTCATCTATATAACAAATTAACAG
AGTCACGTTGATAGAAATGCCGATATTACAGTTTCGTGTGCTGCCGTTGGTGACAAGTGAGTATTATACTTTATTTCATTCTAGAATGGGTG
AATAGAAAGAATCAACTAAGTGGAAACAAAATTACGATTTCCATTGTCTGATTTTCAGCCGCGCATCTGATTATGGATTGGTCAAAGTAGAC
GACAGAGGCAACATCGTACAATTTTCAGAAAAACCGAAAGGCGCTGATCTGAAAGCAATGGTAATTCCAAACATAATAACCTAATGTTTGCA
CCTTCTTTCTGTTTTCTTGTTATCTGGTAAATCCACTGAGATTGTTCATCCTCATTTTGCAGCAAGTAGATACTTCTCGTCTTGGGTTGTCA
CCGCAAGACGCATTGAAGTCGCCATATATTGCATCTATGGGAGTTTATGTGTTCAAGAAAGATGTTTTACTCAAGCTTCTGAAATGGAGGTA
TCCTACTTCTAATGACTTCGGATCCGAAATCATTCCTTCCGCAATAAGAGAGCACAATGTCCAAGTAAGAGGAATTCCGATAAATATATCAG
CTTACAAATGTTTTTATCCATTTCAGAAGATTTTTATCTGCCATCTATGCTTTTATGCAGGCATATTTTTTCGGAGACTACTGGGAAGATAT
TGGAACGATAAAATCCTTCTACGATGCTAACCTCGCTCTTACTGAAGAGGTAGGTTCAAGAATTTTTCAGTGTTCTTGTTCAGTTTAGTTGA
TTGAAACTAAAATCTGCTACATGTTACTCTCTCACAGAGTCCAAAGTTCGAGTTTTATGATCCAAAAACTCCGATTTTCACATCTCCAGGAT
TCCTACCACCAACAAAGATTGACAACTCTCGGGTACGATAATCTATCTATCTTGTATCATGCTAATAATTCGAAACATCATGTCTTTCTTAT
TCTGTCCGCGTTGCTTTGGTTTTGGTAGGTTGTGGATGCCATTATCTCCCATGGATGTTTCCTGAGAGATTGTACAATCCAACACTCCATTG
TAGGTGAAAGGTCGCGTTTAGATTATGGCGTTGAGCTTCAGGTAATTAAACTACCATATATTCTAGACTCGTTACTCCATTAAACGGTTCCT
CCTAACGAAAAACAAGATCAACTTTTACAGGACACTGTAATGATGGGAGCTGACTATTACCAAACTGAATCCGAAATCGCTTCCCTACTTGC
AGAAGGGAAGGTCCCGATTGGCATCGGAAGGAATACCAAAATCAAGTAAAACCTTAGCATTTTCGCGTAGAAAATCTTCCTTTTTACTATTG
TGAAAACTCATAGTTTGTTATTTATTATCTCTGTTAAAGGAACTGCATTATTGACAAGAATGCAAAAATCGGGAAAGAAGTTGTCATCGCGA
ACAAAGAAGTAAGAATAAAACAAGTTAGTAGTTTTCCGTTCCACGATACATTTGTTCACGCGTATAAACTCGAATGCTTTATCTGTAGGGCG
TTCAAGAAGCAGATAGATCGGAAGATGGTTTCTACATCCGATCAGGAATCACCATCATAATGGAGAAAGCAACGATAGAAGACGGAACTGTC
ATATAAACAATGGTTAGTAGTTATTTCACGAGCTGGTTTCCGTAAAGCGCCGGAAGAAGCATTGCAAGGAACACTCCCTCCTATCTTTTGGG
ATTGGTACAAAATGTTATGTTGAATAGAGAAAGCTGCATGTGTAAAATAGGAGAGCTCTTTCACTAGATGTAGAAATAGAAATGAATAAATG
ATGAAAGTGAAGATGCAGAAAAGTTAAATAAATGGAAGGGT

Key:
GCTGCG borders the position of the nine base pair deletion relative to the wild-type gene

INTRON

ATG

TAA predicted by GeneMarker & database comparisons

Ps AGP1L-F1:GCAAGTGCCTTCATTTCTGAG
Ps AGP1L-F2:TGGAGATGGATTTGTGGAGG
Ps AGP1L-F3:GCAAGTAGATACTTCTCGTCTTGGG
Ps AGP1L-F4:ACCGATTTTCACATCTCCTGG
Ps AGP1L-F5:CCTCTTACCAAACGAGCTGC
Ps Agp1l-F6:AGTTTCGTGTGCTGCCG
Ps Agp1l-F7:GAGGTTACTACAATGGCTTCTGG
Ps Agp1l-F8:TTTAGTTGTGACCTTTCACTACTAGC
Ps Agp1l-F9:TTTCTCTCAATAAAGGCAACACC
Ps Agp1l-F10:ACTTCCACATACATCCAAACCC

```
Ps Agp1l-F11:GAATCCGAAATCGCTTCCC
Ps Agp1l-F12:TGGCCGGAGATCATTTATATCG


Ps AGP1L-R1:CTCCTGGTGTTTGAGTCGC
Ps AGP1L-R2:TGAACACATAAACTCCCATAGATGC
Ps AGP1L-R3:CTGAAGCTCAACGCCATAATC
Ps AGP1L-R4:TGATACCTTCACACACTCAACCC
Ps AGP1L-R5:GCATCCTCCAACAGGAACC
Ps Agp1l-R6:ACATTGTGCTCTCTTATTGCGG
Ps Agp1l-R7:GGATAGAGATGTGTTCCAGGC
Ps Agp1l-R8:CGAAATAGGTGCGAGCG
Ps Agp1l-R9:GGAACCTTTGGGGTCATCTG
Ps Agp1l-R10:GCAGGTTTGATCTTCTTCTGGG
Ps Agp1l-R11:CGATGACAACTTCTTTCCCG
Ps Agp1l-R12:GCGAGGTTAGCATCGTAGAAGG
```

Figure S1. The consensus genomic sequence of the naturally occurring *rb* mutant allele for the large subunit of ADP-glucose pyrophosphorylase, present in the pea accession, JI 399. Introns are indicated in yellow highlight; forward and reverse primers are indicated in green and blue font, respectively, and underlined. The position where nine bases are missing relative to the wild-type allele is in pink font. Initiator and stop codons are highlighted in red. The sequences of 12 primer pairs are listed underneath the sequence.

A.

```
JI281Rfs1 --PPSITKTATPQDVINTVDIGNSPLFSISLDQSRNFLVNGHPFLTQVPPNIITTTTSTP     58
JI2110    --PPSITKTATPQDVINTVDIGNSPLFSISLDQSRNFLVNGHPFLTQVPPNITTTTTSTP     58
DB        MAPPSITKTATQQDVISTVDIGNSPLLSISLDQSRNFLVNGHPFLTQVPPNITTTTTSTP     60
            ********* ****.*********:************************* *******

JI281Rfs1 SPFLDFKSNKDTIANNNNTLQQQGCFVGFNTTEAKSHHVVPLGKLKGIKFTSIFRFKVWW     118
JI2110    SPFLDFKSNKDTIANNNNTLQQQGCFVGFNTTEAKSHHVVPLGKLKGIKFTSIFRFKVWW     118
DB        SPFLDFKSNKDTIANNNNTLQQQGCFVGFNTTEAKSHHVVPLGKLKGIKFTSIFRFKVWW     120
          ************************************************************

JI281Rfs1 TTHWVGTNGHELQHETQILILDKNISLGRPYVLLLPILENSFRTSLQPGLNDYVDMSVES     178
JI2110    TTHWVGTNGHELQHETQILILDKNISLGRPYVLLLPILENSFRTSLQPGLNDYVDMSVES     178
DB        TTHWVGTNGHELQHETQILILDKNISLGRPYVLLLPILENSFRTSLQPGLNDYVDMSVES     180
          ************************************************************

JI281Rfs1 GSTHVTGSTFKACLYLHLSNDPYRLVKEAVKVIQTQLGTFKTLEEKTPPSIIEKFGWCTW     238
JI2110    GSTHVTGSTFKACLYLHLSNDPYRLVKEAVKVIQTKLGTFKTLEEKTPPSIIEKFGWCTW     238
DB        GSTHVTGSTFKACLYLHLSNDPYRLVKEAVKVIQTKLGTFKTLEEKTPPSIIEKFGWCTW     240
          ***********************************:************************

JI281Rfs1 DAFYLKVHPKGVREGVKALTDGGCPPGFVIIDDGWQSISHDDDDPVTERDGMNRTSAGEQ     298
JI2110    DAFYLKVHPKGVWEGVKALTDGGCPPGFVIIDDGWQSISHDDDDPVTERDGMNRTSAGEQ     298
DB        DAFYLKVHPKGVWEGVKALTDGGCPPGFVIIDDGWQSISHDDDDPVTERDGMNRTSAGEQ     300
          ************ ***********************************************

JI281Rfs1 MPCRLIKYEENYKFREYENGDNGGKKGLGGFVRDLKEEFRSVESVYVWHALCGYWGGVRP     358
JI2110    MPCRLIKYEENYKFREYENGDNGGKKGLVGFVRDLKEEFRSVESVYVWHALCGYWGGVRP     358
DB        MPCRLIKYEENYKFREYENGDNGGKKGLVGFVRDLKEEFRSVESVYVWHALCGYWGGVRP     360
          **************************** *******************************

JI281Rfs1 KVCGMPEAKVVVPKLSPGMKMTMEDLAVDKIVENGVGLVPPNLAQEMFDGIHSHLESAGI     418
JI2110    KVCGMPEAKVVVPKLSPGVKMTMEDLAVDKIVENGVGLVPPNLAQEMFDGIHSHLESAGI     418
DB        KVCGMPEAKVVVPKLSPGVKMTMEDLAVDKIVENGVGLVPPNLAQEMFDGIHSHLESAGI     420
          ******************:*****************************************

JI281Rfs1 DGVKVDVIHLLELLSEEYGGRVELAKAYYKALTSSVNKHFKGNGVIASMEHCNDFFLLGT     478
JI2110    DGVKVDVIHLLELLSEEYGGRVELAKAYYKALTSSVNKHFKGNGVIASMEHCNDFFLLGT     478
DB        DGVKVDVIHLLELLSEEYGGRVELAKAYYKALTSSVNKHFKGNGVIASMEHCNDFFLLGT     480
          ************************************************************
```

```
JI281Rfs1 EAISLGRVGDDFWCCDPSGDPNGTYWLQGCHMVHCAYNSLWMGNFIHPDWDMFQSTHPCA     538
JI2110    EAISLGRVGDDFWCCDPSGDPNGTYWLQGCHMVHCAYNSLWMGNFIHPDWDMFQSTHPCA     538
DB        EAISLGRVGDDFWCCDPSGDPNGTYWLQGCHMVHCAYNSLWMGNFIHPDWDMFQSTHPCA     540
          ************************************************************

JI281Rfs1 EFHAASRAISGGPVYVSDCVGNHNFKLLKSFVLPDGSILRCQHYALPTRDCLFEDPLHNG     598
JI2110    EFHAASRAISGGPVYVSDCVGNHNFKLLKSFVLPDGSILRCQHYALPTRDCLFEDPLHNG     598
DB        EFHAASRAISGGPVYVSDCVGNHNFKLLKSFVLPDGSILRCQHYALPTRDCLFEDPLHNG     600
          ************************************************************

JI281Rfs1 KTMLKIWNLNKYAGVLGLFNCQGGGWCPETRRNKSASEFSRAVTCYASPEDIEWCNGKTP     658
JI2110    KTMLKIWNLNKYAGVLGLFNCQGGGWCPETRRNKSASEFSHAVTCYASPEDIEWCNGKTP     658
DB        KTMLKIWNLNKYAGVLGLFNCQGGGWCPETRRNKSASEFSHAVTCYASPEDIEWCNGKTP     660
          ****************************************:*******************

JI281Rfs1 MDIKGVDVFAVYFFKEKKLSLMKCSDRLEVSLEPFSFELMTVSPLKVFSKRLIQFAPIGL     718
JI2110    MDIKGVDVFAVYFFKEKKLSLMKCSDRLEVSLEPFSFELMTVSPLKVFSKRLIQFAPIGL     718
DB        MDIKGVDVFAVYFFKEKKLSLMKCSDRLEVSLEPFSFELMTVSPLKVFSKRLIQFAPIGL     720
          ************************************************************

JI281Rfs1 VNMLNSGGAVQSLEFDDSASLVKIGVRGCGELSVFMSEKPVCCKIDGVSVEFDYEDKM--     776
JI2110    VNMLNSGGAVQSLEFDDSASLVKIGVRGCGELSVFASEKPVCCKIDGVSVEFDYEDKM--     776
DB        VNMLNSGGAVQSLEFDDSASLVKIGVRGCGELSVFASEKPVCCKIDGVSVEFDYEDKMVR     780
          *********************************** **********************

JI281Rfs1 ------------------     776
JI2110    ------------------     776
DB        VQILWPGSSTLSLVEFLF     798
```

B.

```
JI281AAP1  MVVEKNASKNHHHQTFDVSIDQQLDSKFFDDDGRVKRTGTSWTASAHVITAVIGSGVLSL     60
JI2110AAP1 MVVEKNASKNHHHQTFDVSIDQQLDSNFFDDDGRVKRTGTSWTASAHVITAVIGSGVLSL     60
AAX56951.1 MVVEKNASKNHHHQTFDVSIDQQLDSKFFDDDGRVKRTGTSWTASAHVITAVIGSGVLSL     60
           **************************:*********************************

JI281AAP1  AWAIAQLGWIAGPVVMILFAWVTYYTSVLLAECYRNGDPVNGKRNYTYMEVVHSNLGGLQ     120
JI2110AAP1 AWAIAQLGWIAGPVVMILFAWVTYYTSVLLAECYRNGDPVNGKRNYTYMEVVHSNLGGLQ     120
AAX56951.1 AWAIAQLGWIAGPVVMILFAWVTYYTSVLLAECYRNGDPVNGKRNYTYMEVVHSNLGGLQ     120
           ************************************************************

JI281AAP1  VQFCGFIQYLNLIGVAIGYTVASAISMMAIERSNCYHRSGGKDPCHMNSNAYMIAFGAVQ     180
JI2110AAP1 VQFCGFIQYLNLIGVAIGYTVASAISMMAIERSNCYHRSGGKDPCHMNSNAYMIAFGAVQ     180
AAX56951.1 VQFCGFIQYLNLIGVAIGYTVASAISMMAIERSNCYHRSGGKDPCHMNSNAYMIAFGAVQ     180
           ************************************************************

JI281AAP1  IIVSQIPDFDQLWWLSIVAAVMSFTYSTIGLGLGIGKVIENKKFAGTITGVNDVTKAQKT     240
JI2110AAP1 IIVSQIPDFDQLWWLSIVAAVMSFTYSTIGLGLGIGKVIENKKFAGTITGVNDVTKAQKT     240
AAX56951.1 IIVSQIPDFDQLWWLSIVAAVMSFTYSTIGLGLGIGKVMENKKFAGTITGVNDVTKAQKT     240
           **************************************:*********************

JI281AAP1  WGSLQALGDIAFAYSFSMILIEIQDTVKAPPPSESKTMKKATLISVIVTTFFYMLCGCLG     300
JI2110AAP1 WGSLQALGDIAFAYSFSMILIEIQDTVKAPPPSESKTMKKATLISVIVTTFFYMLCGCLG     300
AAX56951.1 WGSLQALGDIAFAYSFSMILIEIQDTVKAPPPSESKTMKKATLISVIVTTFFYMLCGCLG     300
           ************************************************************

JI281AAP1  YAAFGNSSPGNLLTGFGFYNPFWLLDIANAAIVIHLIGAYQVYCQPLYAFVENYMTKKFP     360
JI2110AAP1 YAAFGNSSPGNLLTGFGFYNPFWLLDIANAAIVIHLIGAYQVYCQPLYAFVENYMVKRFP     360
AAX56951.1 YAAFGNSSPGNLLTGFGFYNPFWLLDIANAAIVIHLIGAYQVYCQPLYAFVENYMVKRFP     360
           *******************************************************.*:**

JI281AAP1  DNYFLNKNIKIPIPGLDRYKLNLFKLVWRTVFVILTTLVSMLLPFFNDIVGLLGALGFWP     420
JI2110AAP1 DNYFLNKNIKIPIPGLDMYKLNLFKLVWRTVFVILTTLVSMLLPFFNDIVGLLGALGFWP     420
AAX56951.1 DNYFLNKNIKIPIPGLDMYKLNLFKLVWRTVFVILTTLVSMLLPFFNDIVGLLGALGFWP     420
           ***************** ******************************************
```

```
JI281AAP1  LTVYFPVEMYIIQKKIPKWSTKWICLQLLSGACLIITIAASVGSIAGIYLDLKVFKPFKT    480
JI2110AAP1 LTVYFPVEMYIIQKKIPKWSTKWTCLQLLSGACLIITIAASVGSIAGIYLDLKVFKPFKT    480
AAX56951.1 LTVYFPVEMYIIQKKIPKWSTKWTCLQLLSGACLIITIAASVGSIAGIYLDLKVFKPFKT    480
           *********************** ************************************

JI281AAP1  IY   482
JI2110AAP1 IY   482
AAX56951.1 IY   482
           **
```

Figure S2. The protein sequences predicted for genes (*Rfs*, *AAP1*) on LG III which map in the region of the testa trait in JI 2110 x JI 281. **A.** Rfs comparison in JI 2110, JI 281 and the NCBI database accession AJ426475 (DB). **B.** AAP1 comparison in JI 2110, JI 281 and the NCBI accession AAX56951. Asterisks or dots underneath the alignments indicate amino acid identity or extent of similarity, respectively.
