# Supplementary material for: Genetic Variation Controlling Wrinkled Seed Phenotypes in Pisum: How Lucky Was Mendel?
Source: Int J Mol Sci. 2017 Jun 6;18(6):1205. doi: 10.3390/ijms18061205 (PMC5486028; doi:10.3390/ijms18061205)
Supplement: Supplementary file 1 [file ijms-18-01205-s001.zip › ijms-196391-supplementary/ijms-196391-supplefigures-corrected-060617.docx]

Genetic Variation Controlling Wrinkled Seed Phenotypes in *Pisum*: How Lucky Was Mendel?

Tracey Rayner ^1,^*, Carol Moreau1 *, Mike Ambrose ^1^, Peter G. Isaac ^2^, Noel Ellis ^3^ and
Claire Domoney ^1,^*

AAATATAAATTCTAATATGATGCTACCATGATTTATGAAACTCAATCAACCTATCCTATAATCATTAATGTTGTTTACAAATTTTGCAACATGACAAATGACTTCCACATACATCCAAACCCCGCCATCAAATGTCAGATGTAAGCATCCTTAAATCATGAACCAGTAATTTTTTGTTACATAAGATAAAACTACAAATACTTCCCTTTTAAACAACACTTTATTTTTGACTGAAAGTTCTAACACTTTGTTACATTTTTACAACACATTGAAGCAAATAACAACCGCAAAAGTTCACATCAAGTGAAAATGACATTGTCTTTGTTGTGTAATGAGAATCCCATTACAACCTTGGACTTTTGTGAGTGAATCATTTCATCAACCATGTTTCCAAAGGCAAACAAATATCAAAAAATATAATAATATTTTTTTTAGAAATTGAGGTTTCTGATTTCTCTCAATAAAGGCAACACCTTTTCCTTCATCACTACTCAATAATTAGTTTTAGATTCACATTCTTCTCTTTGTTTCTCTAGATTCTATATTTTAGTTGTGACCTTTCACTACTAGCATTGTTTCTCTCTTATTCTCTTGGTCTGAGTTTGAACAAACTCAAAAAAAAAGCTTAGTTTTTTGAGGTTACTACAATGGCTTCTGGTTGTGTGAGCTTGAAAACCAACACCCATTTTCCAAATTCTAAAAAAGGTTCTTTTTTTGGGGAAAGAATCAAAGGAAGCTTGAAAAACAGTTCATGGGTCACTACCCAGAAGAAGATCAAACCTGCTTCTTTTTCTGCTATTCTTACTTCAGATGACCCCAAAGGTTCCCTG*GTAAACTCAGTTTCATTCTGGGTTTCACTTTTTGCTTCCAATTCTGAAAAAAAGAAAGACTTTTTTTTTCTCCCATTATATGACATAACTTTTTTATGTTAATTATTTTGCTACATTTTGTTTGGTATATGATTATGATTATGATTATGATTTTGAGTGTATGTTTTGAAATTCAG*AATTTGCAAGTGCCTTCATTTCTGAGACTAAGAGCTGATCCAAAAAATGTGATTTCCATTGTGTTGGGAGGAGGGCCTGGAACACATCTCTATCCTCTTACCAAACGAGCTG**CG***GTGAGTGAGTTTTATGATATGAATTGTTGTCTGAATTCTAACATTTTGGTGTTGTTTGTAACTTTGTGCTGTTTGTGATGAAGATTTGTTTGCTGTGTTTGTTTTGAAG*GTTCCTGTTGGAGGATGCTATAGGCTTATAGACATTCCAATGAGCAACTGCATCAATAGTGGCATCAACAAGATATTTGTGCTGACTCAGTTCAACTCTGCTTCACTAAATCGTCACATCGCTCGCACCTATTTCGGAAATGGTGTCAACTTTGGAGATGGATTTGTGGAG*GTAATCATGCATCACCATCATGAAAATGGATGGAAATTAGTACTTTTTTTTTCTCGCATTTTTTATATTGGAACAATCTTTTAGCATACTTTGTTTATCTTTTGGTCGAACTCCGAATAACTAGGATCCGTCTTTTCTGGGATTTAGAGAGTTAAGAACAAAAAAATATATTTTCATGCAATGAGTTTTGAGATGAACATGACAGTTGCCAACAAACAAAACATAAGTAATTCGGTTTGCGTAGAGAAATACCGATCCAGTGAAGGAGATTCTTGTTGATGAATTTCAAGTTTGTTTCTATTTATGATTCATGAGGACTAATTAAAGTCAATCTAAATGTCAAAATAG*GTTCTGGCGGCGACACAAACACCAGGAGAAGCTGGGAAGAAGTGGTTTCAAGGAACTGCAGATGCTGTGAGACAATTTACCTGGATATTTGAG*GTAGACAAAGGATTTTTGTTGTTGTTGTATATACATTTCGATAAATAATAGATTCTTTTGTTCTCATTTTTGAGCTTGTCAATAAGTAATAGATTGTTTGTGGTAG*GATGCCAAGAATATAAACGTCGAGAATGTATTGATCTTGGCCGGAGATCATTTATATCGAATGGATTACATGGATCTATTGCAG*GTATATTGTGAATGTTTTGTAGAGTGGATTGTTTTTCATTTCATGTTCTAGAGTTTTCTGATTCATCTATATAACAAATTAACAG*AGTCACGTTGATAGAAATGCCGATATTACAGTTTCGTGTGCTGCCGTTGGTGACAA*GTGAGTATTATACTTTATTTCATTCTAGAATGGGTGAATAGAAAGAATCAACTAAGTGGAAACAAAATTACGATTTCCATTGTCTGATTTTCAG*CCGCGCATCTGATTATGGATTGGTCAAAGTAGACGACAGAGGCAACATCGTACAATTTTCAGAAAAACCGAAAGGCGCTGATCTGAAAGCAATG*GTAATTCCAAACATAATAACCTAATGTTTGCACCTTCTTTCTGTTTTCTTGTTATCTGGTAAATCCACTGAGATTGTTCATCCTCATTTTGCAG*CAAGTAGATACTTCTCGTCTTGGGTTGTCACCGCAAGACGCATTGAAGTCGCCATATATTGCATCTATGGGAGTTTATGTGTTCAAGAAAGATGTTTTACTCAAGCTTCTGAAATGGAGGTATCCTACTTCTAATGACTTCGGATCCGAAATCATTCCTTCCGCAATAAGAGAGCACAATGTCCAA*GTAAGAGGAATTCCGATAAATATATCAGCTTACAAATGTTTTTATCCATTTCAGAAGATTTTTATCTGCCATCTATGCTTTTATGCAG*GCATATTTTTTCGGAGACTACTGGGAAGATATTGGAACGATAAAATCCTTCTACGATGCTAACCTCGCTCTTACTGAAGAG*GTAGGTTCAAGAATTTTTCAGTGTTCTTGTTCAGTTTAGTTGATTGAAACTAAAATCTGCTACATGTTACTCTCTCACAG*AGTCCAAAGTTCGAGTTTTATGATCCAAAAACTCCGATTTTCACATCTCCAGGATTCCTACCACCAACAAAGATTGACAACTCTCGG*GTACGATAATCTATCTATCTTGTATCATGCTAATAATTCGAAACATCATGTCTTTCTTATTCTGTCCGCGTTGCTTTGGTTTTGGTAG*GTTGTGGATGCCATTATCTCCCATGGATGTTTCCTGAGAGATTGTACAATCCAACACTCCATTGTAGGTGAAAGGTCGCGTTTAGATTATGGCGTTGAGCTTCAG*GTAATTAAACTACCATATATTCTAGACTCGTTACTCCATTAAACGGTTCCTCCTAACGAAAAACAAGATCAACTTTTACAG*GACACTGTAATGATGGGAGCTGACTATTACCAAACTGAATCCGAAATCGCTTCCCTACTTGCAGAAGGGAAGGTCCCGATTGGCATCGGAAGGAATACCAAAATCAA*GTAAAACCTTAGCATTTTCGCGTAGAAAATCTTCCTTTTTACTATTGTGAAAACTCATAGTTTGTTATTTATTATCTCTGTTAAAG*GAACTGCATTATTGACAAGAATGCAAAAATCGGGAAAGAAGTTGTCATCGCGAACAAAGAA*GTAAGAATAAAACAAGTTAGTAGTTTTCCGTTCCACGATACATTTGTTCACGCGTATAAACTCGAATGCTTTATCTGTAG*GGCGTTCAAGAAGCAGATAGATCGGAAGATGGTTTCTACATCCGATCAGGAATCACCATCATAATGGAGAAAGCAACGATAGAAGACGGAACTGTCATATAAACAATGGTTAGTAGTTATTTCACGAGCTGGTTTCCGTAAAGCGCCGGAAGAAGCATTGCAAGGAACACTCCCTCCTATCTTTTGGGATTGGTACAAAATGTTATGTTGAATAGAGAAAGCTGCATGTGTAAAATAGGAGAGCTCTTTCACTAGATGTAGAAATAGAAATGAATAAATGATGAAAGTGAAGATGCAGAAAAGTTAAATAAATGGAAGGGT

Key:

**GCTGCG** borders the position of the nine base pair deletion relative to the wild-type gene

*INTRON*

ATG

TAA predicted by GeneMarker & database comparisons

Ps AGP1L-F1:GCAAGTGCCTTCATTTCTGAG

Ps AGP1L-F2:TGGAGATGGATTTGTGGAGG

Ps AGP1L-F3:GCAAGTAGATACTTCTCGTCTTGGG

Ps AGP1L-F4:ACCGATTTTCACATCTCCTGG

Ps AGP1L-F5:CCTCTTACCAAACGAGCTGC

Ps Agp1l-F6:AGTTTCGTGTGCTGCCG

Ps Agp1l-F7:GAGGTTACTACAATGGCTTCTGG

Ps Agp1l-F8:TTTAGTTGTGACCTTTCACTACTAGC

Ps Agp1l-F9:TTTCTCTCAATAAAGGCAACACC

Ps Agp1l-F10:ACTTCCACATACATCCAAACCC

Ps Agp1l-F11:GAATCCGAAATCGCTTCCC

Ps Agp1l-F12:TGGCCGGAGATCATTTATATCG

Ps AGP1L-R1:CTCCTGGTGTTTGAGTCGC

Ps AGP1L-R2:TGAACACATAAACTCCCATAGATGC

Ps AGP1L-R3:CTGAAGCTCAACGCCATAATC

Ps AGP1L-R4:TGATACCTTCACACACTCAACCC

Ps AGP1L-R5:GCATCCTCCAACAGGAACC

Ps Agp1l-R6:ACATTGTGCTCTCTTATTGCGG

Ps Agp1l-R7:GGATAGAGATGTGTTCCAGGC

Ps Agp1l-R8:CGAAATAGGTGCGAGCG

Ps Agp1l-R9:GGAACCTTTGGGGTCATCTG

Ps Agp1l-R10:GCAGGTTTGATCTTCTTCTGGG

Ps Agp1l-R11:CGATGACAACTTCTTTCCCG

Ps Agp1l-R12:GCGAGGTTAGCATCGTAGAAGG

**Figure S1.** The consensus genomic sequence of the naturally occurring *rb* mutant allele for the large subunit of ADP-glucose pyrophosphorylase, present in the pea accession, JI 399. Introns are indicated in yellow highlight; forward and reverse primers are indicated in green and blue font, respectively, and underlined. The position where nine bases are missing relative to the wild-type allele is in pink font. Initiator and stop codons are highlighted in red. The sequences of 12 primer pairs are listed underneath the sequence.

**A.**

JI281Rfs1 --PPSITKTATPQDVINTVDIGNSPLFSISLDQSRNFLVNGHPFLTQVPPNIITTTTSTP 58

JI2110 --PPSITKTATPQDVINTVDIGNSPLFSISLDQSRNFLVNGHPFLTQVPPNITTTTTSTP 58

DB MAPPSITKTATQQDVISTVDIGNSPLLSISLDQSRNFLVNGHPFLTQVPPNITTTTTSTP 60

********* ****.*********:************************* *******

JI281Rfs1 SPFLDFKSNKDTIANNNNTLQQQGCFVGFNTTEAKSHHVVPLGKLKGIKFTSIFRFKVWW 118

JI2110 SPFLDFKSNKDTIANNNNTLQQQGCFVGFNTTEAKSHHVVPLGKLKGIKFTSIFRFKVWW 118

DB SPFLDFKSNKDTIANNNNTLQQQGCFVGFNTTEAKSHHVVPLGKLKGIKFTSIFRFKVWW 120

************************************************************

JI281Rfs1 TTHWVGTNGHELQHETQILILDKNISLGRPYVLLLPILENSFRTSLQPGLNDYVDMSVES 178

JI2110 TTHWVGTNGHELQHETQILILDKNISLGRPYVLLLPILENSFRTSLQPGLNDYVDMSVES 178

DB TTHWVGTNGHELQHETQILILDKNISLGRPYVLLLPILENSFRTSLQPGLNDYVDMSVES 180

************************************************************

JI281Rfs1 GSTHVTGSTFKACLYLHLSNDPYRLVKEAVKVIQTQLGTFKTLEEKTPPSIIEKFGWCTW 238

JI2110 GSTHVTGSTFKACLYLHLSNDPYRLVKEAVKVIQTKLGTFKTLEEKTPPSIIEKFGWCTW 238

DB GSTHVTGSTFKACLYLHLSNDPYRLVKEAVKVIQTKLGTFKTLEEKTPPSIIEKFGWCTW 240

***********************************:************************

JI281Rfs1 DAFYLKVHPKGVREGVKALTDGGCPPGFVIIDDGWQSISHDDDDPVTERDGMNRTSAGEQ 298

JI2110 DAFYLKVHPKGVWEGVKALTDGGCPPGFVIIDDGWQSISHDDDDPVTERDGMNRTSAGEQ 298

DB DAFYLKVHPKGVWEGVKALTDGGCPPGFVIIDDGWQSISHDDDDPVTERDGMNRTSAGEQ 300

************ ***********************************************

JI281Rfs1 MPCRLIKYEENYKFREYENGDNGGKKGLGGFVRDLKEEFRSVESVYVWHALCGYWGGVRP 358

JI2110 MPCRLIKYEENYKFREYENGDNGGKKGLVGFVRDLKEEFRSVESVYVWHALCGYWGGVRP 358

DB MPCRLIKYEENYKFREYENGDNGGKKGLVGFVRDLKEEFRSVESVYVWHALCGYWGGVRP 360

**************************** *******************************

JI281Rfs1 KVCGMPEAKVVVPKLSPGMKMTMEDLAVDKIVENGVGLVPPNLAQEMFDGIHSHLESAGI 418

JI2110 KVCGMPEAKVVVPKLSPGVKMTMEDLAVDKIVENGVGLVPPNLAQEMFDGIHSHLESAGI 418

DB KVCGMPEAKVVVPKLSPGVKMTMEDLAVDKIVENGVGLVPPNLAQEMFDGIHSHLESAGI 420

******************:*****************************************

JI281Rfs1 DGVKVDVIHLLELLSEEYGGRVELAKAYYKALTSSVNKHFKGNGVIASMEHCNDFFLLGT 478

JI2110 DGVKVDVIHLLELLSEEYGGRVELAKAYYKALTSSVNKHFKGNGVIASMEHCNDFFLLGT 478

DB DGVKVDVIHLLELLSEEYGGRVELAKAYYKALTSSVNKHFKGNGVIASMEHCNDFFLLGT 480

************************************************************

JI281Rfs1 EAISLGRVGDDFWCCDPSGDPNGTYWLQGCHMVHCAYNSLWMGNFIHPDWDMFQSTHPCA 538

JI2110 EAISLGRVGDDFWCCDPSGDPNGTYWLQGCHMVHCAYNSLWMGNFIHPDWDMFQSTHPCA 538

DB EAISLGRVGDDFWCCDPSGDPNGTYWLQGCHMVHCAYNSLWMGNFIHPDWDMFQSTHPCA 540

************************************************************

JI281Rfs1 EFHAASRAISGGPVYVSDCVGNHNFKLLKSFVLPDGSILRCQHYALPTRDCLFEDPLHNG 598

JI2110 EFHAASRAISGGPVYVSDCVGNHNFKLLKSFVLPDGSILRCQHYALPTRDCLFEDPLHNG 598

DB EFHAASRAISGGPVYVSDCVGNHNFKLLKSFVLPDGSILRCQHYALPTRDCLFEDPLHNG 600

************************************************************

JI281Rfs1 KTMLKIWNLNKYAGVLGLFNCQGGGWCPETRRNKSASEFSRAVTCYASPEDIEWCNGKTP 658

JI2110 KTMLKIWNLNKYAGVLGLFNCQGGGWCPETRRNKSASEFSHAVTCYASPEDIEWCNGKTP 658

DB KTMLKIWNLNKYAGVLGLFNCQGGGWCPETRRNKSASEFSHAVTCYASPEDIEWCNGKTP 660

****************************************:*******************

JI281Rfs1 MDIKGVDVFAVYFFKEKKLSLMKCSDRLEVSLEPFSFELMTVSPLKVFSKRLIQFAPIGL 718

JI2110 MDIKGVDVFAVYFFKEKKLSLMKCSDRLEVSLEPFSFELMTVSPLKVFSKRLIQFAPIGL 718

DB MDIKGVDVFAVYFFKEKKLSLMKCSDRLEVSLEPFSFELMTVSPLKVFSKRLIQFAPIGL 720

************************************************************

JI281Rfs1 VNMLNSGGAVQSLEFDDSASLVKIGVRGCGELSVFMSEKPVCCKIDGVSVEFDYEDKM-- 776

JI2110 VNMLNSGGAVQSLEFDDSASLVKIGVRGCGELSVFASEKPVCCKIDGVSVEFDYEDKM-- 776

DB VNMLNSGGAVQSLEFDDSASLVKIGVRGCGELSVFASEKPVCCKIDGVSVEFDYEDKMVR 780

*********************************** **********************

JI281Rfs1 ------------------ 776

JI2110 ------------------ 776

DB VQILWPGSSTLSLVEFLF 798

**B.**

JI281AAP1 MVVEKNASKNHHHQTFDVSIDQQLDSKFFDDDGRVKRTGTSWTASAHVITAVIGSGVLSL 60

JI2110AAP1 MVVEKNASKNHHHQTFDVSIDQQLDSNFFDDDGRVKRTGTSWTASAHVITAVIGSGVLSL 60

AAX56951.1 MVVEKNASKNHHHQTFDVSIDQQLDSKFFDDDGRVKRTGTSWTASAHVITAVIGSGVLSL 60

**************************:*********************************

JI281AAP1 AWAIAQLGWIAGPVVMILFAWVTYYTSVLLAECYRNGDPVNGKRNYTYMEVVHSNLGGLQ 120

JI2110AAP1 AWAIAQLGWIAGPVVMILFAWVTYYTSVLLAECYRNGDPVNGKRNYTYMEVVHSNLGGLQ 120

AAX56951.1 AWAIAQLGWIAGPVVMILFAWVTYYTSVLLAECYRNGDPVNGKRNYTYMEVVHSNLGGLQ 120

************************************************************

JI281AAP1 VQFCGFIQYLNLIGVAIGYTVASAISMMAIERSNCYHRSGGKDPCHMNSNAYMIAFGAVQ 180

JI2110AAP1 VQFCGFIQYLNLIGVAIGYTVASAISMMAIERSNCYHRSGGKDPCHMNSNAYMIAFGAVQ 180

AAX56951.1 VQFCGFIQYLNLIGVAIGYTVASAISMMAIERSNCYHRSGGKDPCHMNSNAYMIAFGAVQ 180

************************************************************

JI281AAP1 IIVSQIPDFDQLWWLSIVAAVMSFTYSTIGLGLGIGKVIENKKFAGTITGVNDVTKAQKT 240

JI2110AAP1 IIVSQIPDFDQLWWLSIVAAVMSFTYSTIGLGLGIGKVIENKKFAGTITGVNDVTKAQKT 240

AAX56951.1 IIVSQIPDFDQLWWLSIVAAVMSFTYSTIGLGLGIGKVMENKKFAGTITGVNDVTKAQKT 240

**************************************:*********************

JI281AAP1 WGSLQALGDIAFAYSFSMILIEIQDTVKAPPPSESKTMKKATLISVIVTTFFYMLCGCLG 300

JI2110AAP1 WGSLQALGDIAFAYSFSMILIEIQDTVKAPPPSESKTMKKATLISVIVTTFFYMLCGCLG 300

AAX56951.1 WGSLQALGDIAFAYSFSMILIEIQDTVKAPPPSESKTMKKATLISVIVTTFFYMLCGCLG 300

************************************************************

JI281AAP1 YAAFGNSSPGNLLTGFGFYNPFWLLDIANAAIVIHLIGAYQVYCQPLYAFVENYMTKKFP 360

JI2110AAP1 YAAFGNSSPGNLLTGFGFYNPFWLLDIANAAIVIHLIGAYQVYCQPLYAFVENYMVKRFP 360

AAX56951.1 YAAFGNSSPGNLLTGFGFYNPFWLLDIANAAIVIHLIGAYQVYCQPLYAFVENYMVKRFP 360

*******************************************************.*:**

JI281AAP1 DNYFLNKNIKIPIPGLDRYKLNLFKLVWRTVFVILTTLVSMLLPFFNDIVGLLGALGFWP 420

JI2110AAP1 DNYFLNKNIKIPIPGLDMYKLNLFKLVWRTVFVILTTLVSMLLPFFNDIVGLLGALGFWP 420

AAX56951.1 DNYFLNKNIKIPIPGLDMYKLNLFKLVWRTVFVILTTLVSMLLPFFNDIVGLLGALGFWP 420

***************** ******************************************

JI281AAP1 LTVYFPVEMYIIQKKIPKWSTKWICLQLLSGACLIITIAASVGSIAGIYLDLKVFKPFKT 480

JI2110AAP1 LTVYFPVEMYIIQKKIPKWSTKWTCLQLLSGACLIITIAASVGSIAGIYLDLKVFKPFKT 480

AAX56951.1 LTVYFPVEMYIIQKKIPKWSTKWTCLQLLSGACLIITIAASVGSIAGIYLDLKVFKPFKT 480

*********************** ************************************

JI281AAP1 IY 482

JI2110AAP1 IY 482

AAX56951.1 IY 482

**

**Figure S2.** The protein sequences predicted for genes (*Rfs*, *AAP1*) on LG III which map in the region of the testa trait in JI 2110 x JI 281. **A.** Rfs comparison in JI 2110, JI 281 and the NCBI database accession AJ426475 (DB). **B.** AAP1 comparison in JI 2110, JI 281 and the NCBI accession AAX56951. Asterisks or dots underneath the alignments indicate amino acid identity or extent of similarity, respectively.
